# iPSC-derived blood-brain barrier modeling reveals APOE isoform-dependent interactions with amyloid beta

**DOI:** 10.1186/s12987-024-00580-2

**Published:** 2024-10-11

**Authors:** Yunfeng Ding, Sean P. Palecek, Eric V. Shusta

**Affiliations:** 1 Department of Chemical and Biological Engineering, University of Wisconsin–Madison, 1415 Engineering Drive, Madison, WI 53706 USA; 2 Department of Neurological Surgery, University of Wisconsin–Madison, Madison, WI USA

**Keywords:** Blood-brain barrier, Alzheimer’s disease, Endothelial cells, Pericytes, Disease modeling, Amyloid deposition, Induced pluripotent stem cells, Apolipoprotein E (APOE)

## Abstract

**Background:**

Three common isoforms of the apolipoprotein E (*APOE*) gene - *APOE2*, *APOE3*, and *APOE4* - hold varying significance in Alzheimer’s Disease (AD) risk. The *APOE4* allele is the strongest known genetic risk factor for late-onset Alzheimer’s Disease (AD), and its expression has been shown to correlate with increased central nervous system (CNS) amyloid deposition and accelerated neurodegeneration. Conversely, *APOE2* is associated with reduced AD risk and lower CNS amyloid burden. Recent clinical data have suggested that increased blood-brain barrier (BBB) leakage is commonly observed among AD patients and *APOE4* carriers. However, it remains unclear how different *APOE* isoforms may impact AD-related pathologies at the BBB.

**Methods:**

To explore potential impacts of *APOE* genotypes on BBB properties and BBB interactions with amyloid beta, we differentiated isogenic human induced pluripotent stem cell (iPSC) lines with different *APOE* genotypes into both brain microvascular endothelial cell-like cells (BMEC-like cells) and brain pericyte-like cells. We then compared the effect of different *APOE* isoforms on BBB-related and AD-related phenotypes. Statistical significance was determined via ANOVA with Tukey’s post hoc testing as appropriate.

**Results:**

Isogenic BMEC-like cells with different *APOE* genotypes had similar trans-endothelial electrical resistance, tight junction integrity and efflux transporter gene expression. However, recombinant APOE4 protein significantly impeded the “brain-to-blood” amyloid beta 1–40 (Aβ40) transport capabilities of BMEC-like cells, suggesting a role in diminished amyloid clearance. Conversely, APOE2 increased amyloid beta 1–42 (Aβ42) transport in the model. Furthermore, we demonstrated that APOE-mediated amyloid transport by BMEC-like cells is dependent on LRP1 and p-glycoprotein pathways, mirroring in vivo findings. Pericyte-like cells exhibited similar APOE secretion levels across genotypes, yet *APOE4* pericyte-like cells showed heightened extracellular amyloid deposition, while *APOE2* pericyte-like cells displayed the least amyloid deposition, an observation in line with vascular pathologies in AD patients.

**Conclusions:**

While *APOE* genotype did not directly impact general BMEC or pericyte properties, APOE4 exacerbated amyloid clearance and deposition at the model BBB. Conversely, APOE2 demonstrated a potentially protective role by increasing amyloid transport and decreasing deposition. Our findings highlight that iPSC-derived BBB models can potentially capture amyloid pathologies at the BBB, motivating further development of such in vitro models in AD modeling and drug development.

**Supplementary Information:**

The online version contains supplementary material available at 10.1186/s12987-024-00580-2.

## Background

The blood-brain barrier (BBB) is a dynamic interface that separates the bloodstream and the central nervous system (CNS), helping to maintain CNS homeostasis. BBB properties are largely characteristics of brain endothelial cells, that are in turn modulated by other cellular constituents of the neurovascular unit (NVU), such as pericytes, astrocytes, neurons, and perivascular macrophages [1, 2]. In healthy individuals, the BBB safeguards the CNS from deleterious substances by providing both a passive barrier and a host of transporters that combine to regulate the entry and efflux of many small molecules, proteins, and cells [3]. However, in many CNS diseases, the BBB not only provides a significant barrier for the delivery of many neurotherapeutics, but also contributes to disease pathogenesis. For example, increased BBB permeability has also been observed in several neurodegenerative diseases, including Alzheimer’s Disease (AD) and Parkinson’s Disease (PD), but the role of the BBB in the pathophysiology of these diseases is not yet clear [4, 5].

AD is the leading cause of dementia, affecting more than 30 million people worldwide [6, 7]. At the cellular level, synaptic dysfunction, neurodegeneration, inflammation, and vascular dysfunction are common pathological features of AD [8–10]. In recent years, genetic risk factors for AD have been identified, among which alleles of the *APOE* gene are the strongest and most prevalent [7, 11, 12]. *APOE* encodes for apolipoprotein E, a 34 kDa glycoprotein that plays a crucial role in lipid metabolism by binding to cholesterol and phospholipids and facilitating their transport throughout the body [7, 13]. In the periphery, the liver is the predominant producer of APOE [14]. In the CNS, astrocytes, microglia and pericytes have been shown to secrete APOE [15, 16]. There are three dominant isoforms of *APOE*, namely *APOE2*, *APOE3* and *APOE4*, which differ by only two single nucleotide polymorphisms (SNPs) [15]. Among the three isoforms, *APOE3* is the most prevalent in the human population [17]. *APOE4* allele carriers have significantly increased AD risk [18] and the *APOE2* allele is considered a protective isoform against AD [19]. While the exact mechanism by which different *APOE* isoforms contribute to AD pathophysiology remains to be elucidated, human and animal studies have consistently shown that *APOE4* is linked to increased deposition of amyloid beta (Aβ) plaques in the brain [18, 20–25], impaired intracellular lysosomal degradation of Aβ [26–29], and increased levels of neuroinflammation [30–33].

The BBB plays a central role in Aβ trafficking and is thus heavily involved in AD pathogenesis [34–37]. It is estimated that the BBB clears more than 85% of all Aβ produced in the CNS through various pathways [38, 39]. Many studies have demonstrated different aspects of BBB breakdown and dysregulation in AD patients, such as increased hippocampal BBB permeability [40, 41], impaired Aβ efflux from the CNS [42, 43], reduced pericyte coverage of CNS microvessels [44, 45], infiltration of peripheral immune cells into the CNS [46, 47], and reduced tight junction gene expression and basement membrane alterations [48–51]. These BBB pathologies are more pronounced in patients that are *APOE4* carriers [52], including more severe BBB breakdown [21, 53, 54], accelerated degeneration of brain pericytes [55–57], increased amyloid deposition [24], and worsened cognitive decline [58, 59]. Moreover, *APOE4* carriers have lower levels of peripheral blood Aβ and cerebral spinal fluid (CSF) Aβ [60–62], consistent with impaired clearance. Despite the prevalence of vascular pathologies in AD patients, the causal relationship between BBB damage and AD remains a subject of research [63].

To investigate the role of different *APOE* isoforms on BBB function and amyloid deposition and transport, we used established protocols to differentiate isogenic human induced pluripotent stem cells (iPSCs) expressing different *APOE* alleles to brain microvascular endothelial cell-like cells (BMEC-like cells) and brain pericyte-like cells [64–67]. We used commercially available, isogenic iPSC lines that were created with CRISPR/Cas9 genome editing of both *APOE* alleles to produce *APOE KO/KO*,* APOE2/APOE2*, *APOE3/APOE3* and *APOE4/APOE4* lines to minimize the confounding effect of genetic background. We found that iPSC-derived BMEC-like cells and pericyte-like cells with different *APOE* isoforms are similar in terms of BBB-related gene expression and key barrier and transporter phenotypes. However, *APOE2* BMEC-like cells demonstrated improved Aβ42 clearance compared to *APOE3* and *APOE4* counterparts while recombinant APOE4 protein led to decreased Aβ42 clearance compared with APOE2 and APOE3 proteins. In pericyte-like cells, *APOE4* pericyte-like cells had increased extracellular Aβ42 deposition and *APOE2* pericyte-like cells had reduced deposition compared to *APOE3* pericyte-like cells. Collectively, our findings are consistent with clinical observations that *APOE4* leads to higher amyloid load while *APOE2* leads to a lower amyloid load.

## Methods

### Isogenic iPSC differentiation to BMEC-like cells and brain-like Pericytes

Isogenic iPSC lines with different *APOE* genotypes iPS16 (*APOE4/E4*, Alstem, abbreviated as *APOE4* in this manuscript), iPS26 (*APOE3/E3*, Alstem, abbreviated as *APOE3* in this manuscript), iPS36 (*APOE KO/KO*, Alstem, abbreviated as *APOE KO* in this manuscript), iPS46 (*APOE2/E2*, Alstem, abbreviated as *APOE2* in this manuscript) were obtained. iPSCs were cultured between passages 20–50 on Matrigel (Corning, 354277) with daily mTESR1 (StemCell Technologies, 85850) medium changes.

BMEC-like cells were differentiated as previously described [64, 65, 68–70]. In brief, iPSCs were singularized with Accutase (Life Technologies, 00-4555-56) and expanded to 30,000 cells/cm^2^ prior to the initiation of the differentiation. Unconditioned medium (UM), which is prepared by mixing 100 mL Knock-out serum replacement (Life Technologies, 10828028), 5 mL MEM non-essential amino acids solution (Life Technologies, 11140050), 2.5 mL of GlutaMAX (Life Technologies, 35050061), 392.5 mL of DMEM/F12 (Life Technologies, 11320033) and 3.5 µL of β-mercapto-ethanol (Sigma, 444203), was changed daily for 6 days. UM was changed to hECSR medium, which is human Endothelial Serum-Free medium (Life Technologies, 11111044), 20 ng/mL basic fibroblast growth factor (Peprotech, 100-18B) and 2% B-27 (Life Technologies, 17504044), for 48 h supplemented with 10 µM retinoic acid (StemCell Technologies, 72264).

Pericytes were derived in a two-step process as described previously [67, 70]. Initially iPSCs were differentiated in Essential 6 medium (Life Technologies, A1516401) supplemented with 1 μm dorsomorphin (StemCell Technologies, 72102), 10 μm SB431542 (Tocris, 1614), 1 μm CHIR99021 (Tocris, 4953), 10 ug/L basic fibroblast growth factor (Peprotech, 100-18B), and 22.5 mg/L heparin (Sigma, H3393) to a neural-crest stem cell population. CD271 + cells were enriched with neural crest stem cell microbeads (Miltenyi, 130-097-127) and seeded at 10,000 cells/cm^2^ onto tissue culture-treated plates. Following 6 days of expansion in Essential 6 medium (Life Technologies, A1516401) supplemented with 10% FBS (Life Technologies, A5256701), pericytes were harvested for analysis.

### Aβ transcytosis assays

Transwells (Corning, 3460) pre-coated with 200 uL 0.5 mg/mL collagen IV (Sigma, C5533) overnight at 37 °C. iPSC-derived BMEC-like cells were seeded at 1 million/cm^2^ on Transwells (Corning, 3460) in hECSR media. Recombinant human Aβ40 (Anaspec, AS-24235) or recombinant human Aβ42 (Anaspec, AS20276) were prepared in 8 M urea 100 mM glycine buffer at pH = 10 to a concentration of 12 mg/mL and stored in aliquots at -80 °C. This buffer helps to maintain Aβ in monomeric and denatured state. Stocks were then diluted to 500 nM in hECSR medium immediately before experiments. For experiments conditions when recombinant APOE is added, recombinant APOE2 (Peprotech, 350 − 12), APOE3 (Peprotech, 350-02) or APOE4 (Peprotech, 350-04) were added at concentration of 500 nM. In transcytosis assay, 1.5 mL of hECSR medium with Aβ and/or recombinant APOE was added to the basolateral chamber. 0.5 mL of hECSR media that is Aβ-free and APOE-free was added to the apical chamber. The Transwells were then incubated at 37 °C on a 300 rpm orbital shaker for 3 h. Media samples from the apical chamber were taken and analyzed. To quantify Aβ concentrations in the media samples, ELISA kits for Aβ40 (Life Technologies, KHB3481) or Aβ42 (Life Technologies, KHB3441) were used.

### Trans-endothelial electrical resistance (TEER) measurements

EVOM 2 with STX4 electrodes (World Precision Instruments) were used to measure the TEER value in Ω after BMEC differentiation and seeding on Transwells on Day 10. The values are normalized by subtracting the TEER reading from a collagen IV-coated blank well and then multiplied by the surface area of the Transwell insert and reported as Ω∙cm^2^.

### P-glycoprotein efflux transporter assay

BMEC-like cells were seeded on collagen IV-coated plates at 100,000 cells/cm^2^. Half of the wells of each experimental group were treated with 10 μm Cyclosporin A (CsA, Tocris, 1101) for 30 min in hECSR medium, with the other half treated with DMSO (Sigma, D2650) in hECSR. After incubation, a solution of 10 μm rhodamine 123 (Rh123, Life Technologies, R302) in hECSR with and without CsA, was added to corresponding wells. The plates were kept at 37 °C for one hour at 300 rpm on an orbital shaker. After incubation, cells were washed three times with ice cold DPBS (Life Technologies, 14040117) and then lysed with RIPA buffer (Life Technologies, 89901). We used a fluorescent plate reader to measure the fluorescence of Rh123 accumulated inside the cells. We used a BCA assay kit (Life Technologies, 23225) to quantify total protein from cell lysates. The fluorescence readings were normalized to total protein concentrations of the lysates.

### Immunocytochemistry

Cells were fixed with − 20 °C methanol (Sigma, 67-56-1) or 4% paraformaldehyde (PFA; Electron Microscopy Sciences, 15700) in DPBS for 15 min at room temperature. Following three washes in DPBS the cells were blocked in 10% goat serum (Sigma, G9023) in PBS for 30 min. Cells were then incubated with primary antibodies at indicated dilution ratios at 4 °C overnight (Table 1). Cells were then washed with PBS three times. Cells were then incubated with secondary antibodies at the indicated dilution ratios (Table 1) and 20 μm Hoechst 33,342 (Thermo Scientific, 62249) for 1 h at room temperature. Cells were then washed three times. For widefield fluorescence microscopy, we used a Nikon Eclipse Ti2 microscope. For confocal microscopy, we treated samples with ProLong Gold Antifade (Life Technologies, P36941) and imaged using a Nikon A1R microscope. For quantification of immunocytochemistry images, background signals from a secondary antibody only control were subtracted from all conditions. Area fraction index was calculated as previously reported [71].

**Table 1:** Antibodies used in this study

Antibody	Source	Dilution ratios
Anti-claudin-5 (mouse monoclonal IgG1, clone 4C3C2)	Invitrogen 35-2500	1:100 (ICC)
Anti-OCLN (mouse monoclonal IgG1, clone OC-3F10)	Invitrogen 33-1500	1:100 (ICC)
Anti-LRP1 (rabbit monoclonal IgG, clone SA0290)	Invitrogen MA5-31959	1:100 (ICC)
Anti-CDH5 (mouse monoclonal IgG2a, clone BV9)	Santa Cruz Biotechnology sc-52,751	1:100 (ICC)
Anti-NG2 (mouse monoclonal IgG2a, clone 9.2.27)	Millipore MAB2029	1:50 (Flow Cytometry)
Anti-PDGFRβ (mouse monoclonal IgG2a, clone 28D4)	BD Biosciences, 558,820	1:100 (Flow Cytometry)
Anti-Aβ (mouse monoclonal IgG2a, clone B-4)	Santa Cruz Biotechnology sc-28,365 FITC	1:100 (Flow Cytometry)
Alexa Fluor 488 goat anti-mouse IgG (goat polyclonal)	Invitrogen A-28,175	1:200 (ICC), 1:200 (Flow Cytometry)
Alexa Fluor 647 goat anti-rabbit IgG (goat polyclonal)	Invitrogen A-21,245	1:200 (ICC), 1:200 (Flow Cytometry)

### Reverse transcription real-time PCR analysis

RNA was extracted using Direct-zol RNA Miniprep kit (Zymo, R2050). Reverse transcription was performed to obtain cDNA using GoScript Reverse Transcriptase with Oligo(dT) kit (Promega, A2791). Real-time gene expression analysis was conducted using 25 µl reactions containing SYBR Green PCR Master Mix (Life Technologies, 4309155) along with primers specific for genes of interest (Table 2). PCR was run according to manufacturer protocols on an Agilent AriaMX Real-Time PCR system.

**Table 2:** Primer sequences for RT-qPCR used in this study

Gene	Forward primer (5’-3’)	Reverse primer (5’-3’)
*CLDN5*	TGACCTTCTCCTGCCACTA	AAGCGAAATCCTCAGTCTGAC
*OCLN*	ATGGCAAAGTGAATGACAAGC	AGGCGAAGTTAATGGAAGCTC
*TJP1*	CGCGTCTCTCCACATACATTC	GCTGGCTTATTCTGAGATGGA
*SLC2A1*	GTGCCATACTCATGACCATCG	GGCCACAAAGCCAAAGATG
*ABCB1*	ACTCACTTCAGGAAGCAACC	GATTGACTGAATGCTGATTCCTC
*ABCG2*	CTCAGATCATTGTCACAGTCGT	GTCGTCAGGAAGAAGAGAACC
*LRP1*	TCCAGTACAGATTGTCTCCCA	ATCTACTTTGCCGACACCAC
*AGER*	ATTCTGCCTCTGAACTCACG	TCCTTCACAGATACTCCCTTCT
*APOE*	GGGTCGCTTTTGGGATTACCTG	CAACTCCTTCATGGTCTCGTCC

### Flow cytometry-based Aβ deposition analysis

After differentiation, pericyte-like cells were treated with Essential 6 medium (Life Technologies, A1516401) with or without 500nM Aβ42 (Anaspec, AS20276). Cells were incubated for 18 h at 37 °C on a 300 rpm orbital shaker. The extended incubation time allowed Aβ42 to interact with pericyte-like cell-secreted APOE, aggregate, and form extracellular amyloid deposits. After incubation, pericyte-like cells were treated with Accutase (Life Technologies, 00-4555-56) for 7 min to singularize the cells. Cells were removed from the plate, washed three times with cold PBS, and fixed with 4% PFA (Electron Microscopy Sciences, 15700). Fixed pericyte-like cells were then incubated with Alexa Fluor 488-conjugated anti-Aβ antibody (Biolegend, 6E10) at 1:100 dilution ratio in eBioscience Flow Cytometry Staining Buffer (Life Technologies, 00-4222-26) for 1 h. Pericyte-like cells were then washed three times with cold PBS, and analyzed on a BD Accuri C6 flow cytometer.

### Statistics

Biological replicates in this manuscript refer to individual wells of cultured cells that underwent identical experimental treatments. The authors of the study ensured that all key experiments were repeated using multiple independent iPSC differentiations from the isogenic iPSC lines. The replication strategy for each experiment is stated in figure legends. To compare means of two experimental groups, Student’s t test was used. For experiments with three or more experimental groups, one-way analysis of variance (ANOVA) was used for comparison of means. For experiments with two factors of experimental conditions, two-way ANOVA was used. Following ANOVA, Dunnett’s post hoc test was used for comparison of multiple treatments to a single control, or Tukey’s honest significant difference test was used for multiple pairwise comparisons. Statistical tests were performed in GraphPad Prism. Descriptions of the statistical tests used are provided in figure legends.

## Results

### iPSC-derived BMEC-like cells with different *APOE* isoforms have similar barrier and transporter properties

**Fig. 1:**
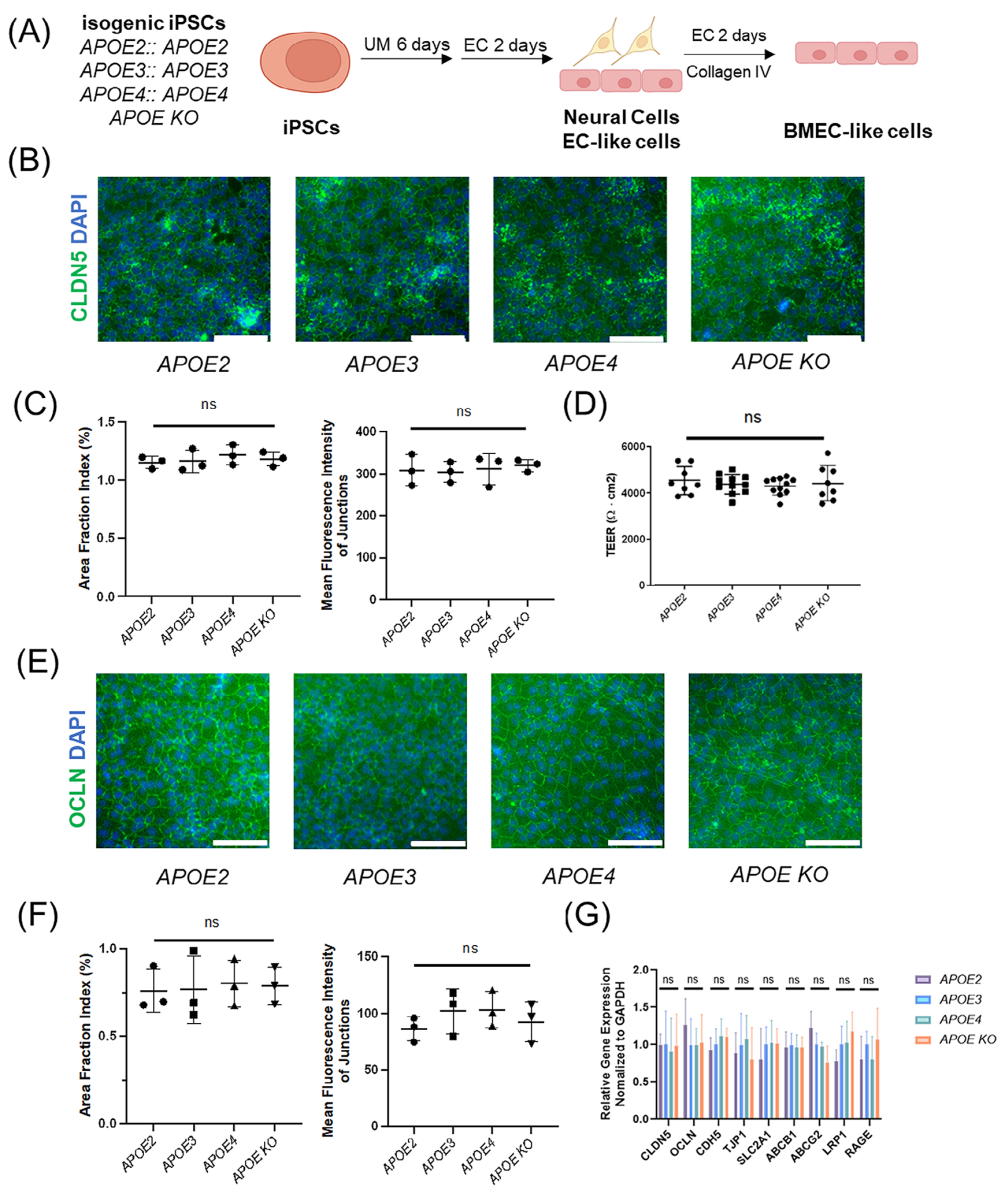
iPSC-derived BMEC-like cells expressing different APOE isoforms have similar barrier and transporter properties. (**A**) Isogenic iPSCs carrying different *APOE* genotypes were differentiated to BMEC-like cells via the UM differentiation method. (**B**) Immunocytochemistry analysis of claudin-5 (CLDN5) expression and nuclear staining by DAPI in Day 10 isogenic iPSC-derived BMEC-like cells. Scale bars: 100 μm. (**C**) Quantification of claudin-5 expression and localization in (**B**) by area fraction index of immunocytochemistry images, which analyzes the fraction of pixels that belong to a junction and the mean fluorescence intensity of the junctions. *n* = 3 independent differentiations for each condition. Data are reported as mean ± standard deviation. ns: p-value > 0.05 in one-way ANOVA analysis. (**D**) Comparison of Day 10 trans-endothelial electrical resistance (TEER) among BMEC-like cells expressing different APOE alleles. Data are reported as mean ± standard deviation. *n* = 8 independent differentiations for each cell line. ns: p-value > 0.05 in one-way ANOVA analysis. (**E**) Immunocytochemistry analysis of occludin (OCLN) expression and nuclear staining by DAPI in Day 10 isogenic iPSC-derived BMEC-like cells. Scale bars: 100 μm. (**F**) Quantification of occludin expression and localization in (**E**) by area fraction index of immunocytochemistry images, which analyzes the fraction of pixels that belong to a junction and the mean fluorescence intensity of the junctions. *n* = 3 independent differentiations for each condition. Data are reported as mean ± standard deviation. ns: p-value > 0.05 in one-way ANOVA analysis. (**G**) RT-qPCR analysis of expression of BBB junction (*CLDN5*, *OCLN*, *CDH5*, *TJP1*) and transporter (*SLC2A1*, *ABCB1*, *ABCG2*, *LRP1*, *RAGE*) genes in Day 10 isogenic BMEC-like cells (*n* = 3 independent differentiations each). Data are normalized to the APOE3 condition for each transcript. Data are reported as mean ± standard deviation. ns: p-value > 0.05 in one-way ANOVA analysis

Established protocols [64, 65, 68] were used to differentiate isogenic iPSC cell lines with homozygous *APOE2*, *APOE3*, *APOE4* or *APOE* Knockout (*APOE KO*) isoforms into BMEC-like cells. Briefly, the iPSCs were differentiated in unconditioned medium (UM) for six days, maintained in endothelial cell (EC) medium for two days and the mixed differentiating cell populations were plated on collagen IV matrix to purify the BMEC-like cells (Fig. 1A). In addition, flow cytometry analysis of endothelial markers claudin-5 and VE-cadherin revealed that isogenic BMEC-like cells, regardless of *APOE* genotypes, are homogenous cell populations (Ext. Figure 1D). The resulting cells possess BMEC-like properties as previously described, including tight junction protein expression (Fig. 1B and E, Ext. Figure 1A), tight barrier function (Fig. 1D, Ext. Figure 1E), and efflux transporter gene expression (Fig. 1G). Immunocytochemistry was used to visualize and quantify the expression and localization of junctional proteins occludin and claudin-5, and we observed no significant differences in junctional area fraction index or mean fluorescence intensity of these proteins at junctions as a function of *APOE* genotype (Fig. 1B, C, E and F). The iPSC-derived BMEC-like cells were also seeded onto Transwells for quantification of trans-endothelial electrical resistance (TEER). We found that BMEC-like cells with different *APOE* genotypes all developed an indistinguishably tight barrier with TEER greater than 3000 Ω∙cm^2^ (Fig. 1D). Monolayers formed by isogenic BMEC-like cells also demonstrate similar permeability to sodium fluorescein (Ext. Figure 1E). By RT-qPCR, we found that BMEC-like cells with different *APOE* genotypes expressed similar levels of *APOE* transcript (Ext. Figure 1B), and the concentration of APOE in cell culture media conditioned by isogenic BMEC-like cells for 24 h was below the limit of detection of ELISA (Ext. Figure 1C), indicating that the APOE protein was not substantially synthesized in BMEC-like cells. Since the *APOE KO* line was generated by introducing a 100 bp homozygous deletion in the exon 2 of *APOE* gene, which is not at the region of our qPCR primers, we were still able to detect mRNA transcripts for *APOE* by qPCR, but APOE protein expression was absent by ELISA. (Ext. Figure 1B and C). We assessed the transcript expression of several canonical BMEC tight junction (*CLDN5*, *OCLN*, *CDH5*, *TJP1*), transporter (*SLC2A1*, *ABCB1*, *ABCG2*) and Aβ-related transporter (*LRP1*, *RAGE*) [72, 73] genes by RT-qPCR and observed no significant differences in BMEC-like cells derived from different *APOE* genotypes (Fig. 1G). Collectively, these data indicate that isogenic iPSC-derived BMEC-like cells with different *APOE* genotypes have similar barrier properties and indistinguishable expression levels of key BMEC genes.

### LRP1 and P-glycoprotein-mediated pathways are involved in Aβ transport by iPSC-derived BMEC-like cells

**Fig. 2:**
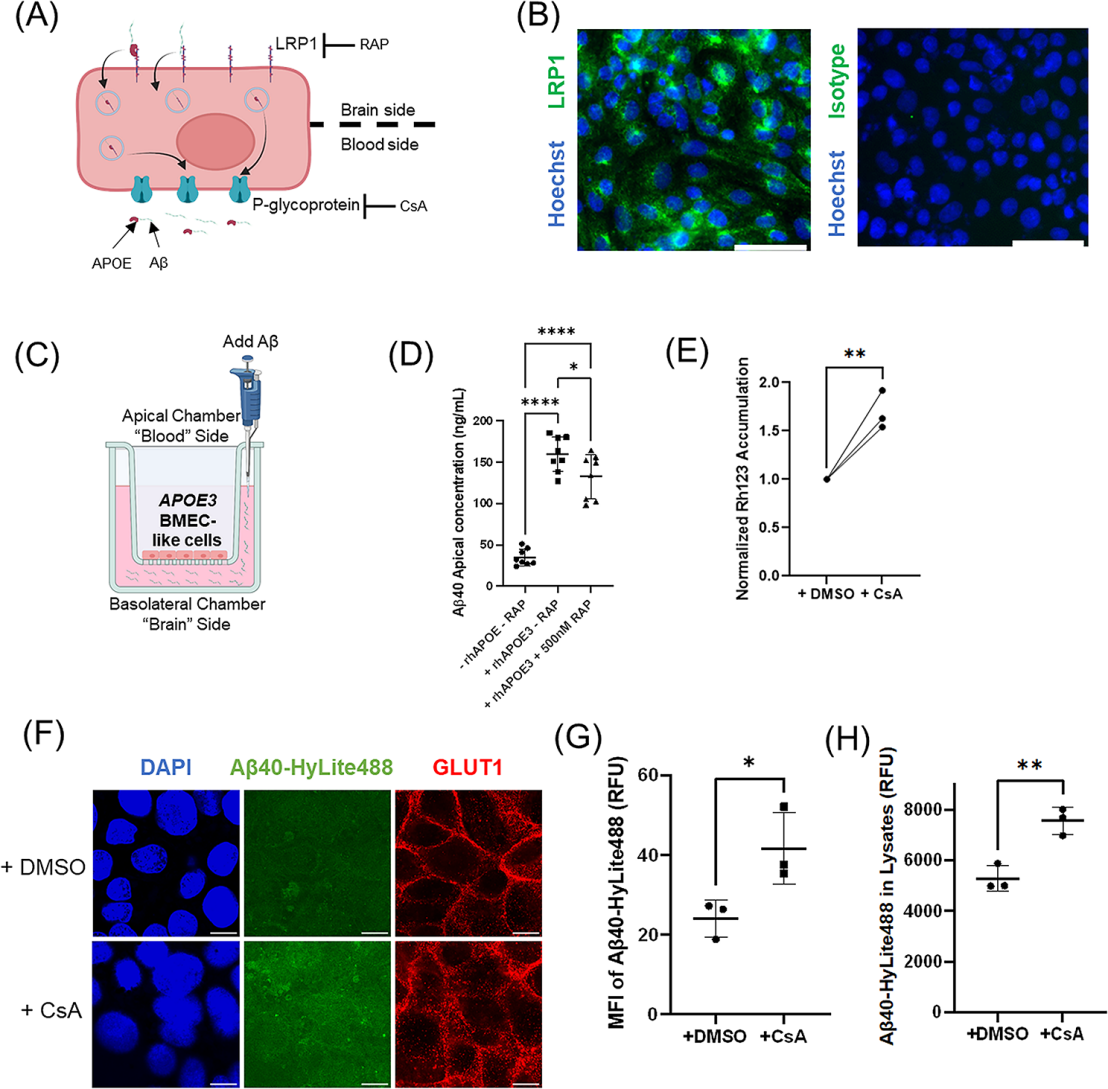
LRP1 and P-glycoprotein-mediated pathways are involved in Aβ transport by iPSC-derived BMEC-like cells. (**A**) Schematic depicting the involvement of LRP1 and p-glycoprotein in Aβ trafficking in BMECs. (**B**) Immunocytochemistry analysis of LRP1 expression, along with isotype control antibody and nuclear staining by Hoechst 33342, in Day 10 *APOE3* iPSC-derived BMEC-like cells Scale bars: 100 μm. (**C**) Schematic showing Transwell experimental design for analyzing brain-to-blood Aβ trafficking in iPSC-derived BMEC-like cells. (**D**) Basolateral-to-apical transport of Aβ40 with and without RAP treatment. Aβ40 was dosed into the basolateral chamber of the Transwell, and apical concentration of Aβ40 was determined by ELISA after 3 h. (*n* = 8 independent wells of BMEC-like cells for each condition) *: *p* < 0.05, ****: *p* < 0.0001 by one-way ANOVA followed by Tukey’s test. Data are reported as mean ± standard deviation. (**E**) Intracellular Rhodamine 123 (Rh123) accumulation with and without CsA treatment across three differentiations of *APOE3* iPSC-derived BMEC-like cells. BMEC-like cells were incubated with Rh123 pretreated with DMSO or CsA. Fluorescence of Rh123 from cell lysates was quantified by plate reader. (*n* = 3 independent biological replicates) Data are normalized to the control DMSO condition for each pair. **: *p* < 0.01 by paired Student’s t-test. (**F**) Representative confocal microscopy images showing accumulation of intracellular Aβ40-Hylite488 inside *APOE3* iPSC-derived BMEC-like cells with and without P-gp inhibition by CsA. Confocal microscopy images were taken at the z-plane where DAPI nucleus staining was visible. Intracellular Aβ40-Hylite488 fluorescence was then quantified in this same z-plane image. GLUT1 can be found both at the cell surface and junctions in BMEC-like cells and its immunolabeling was included to help visualization of the cell junctions at the same z-plane. Scale bars: 10 μm. (**G**) Quantification of the mean fluorescence intensity of Aβ40-Hylite488 accumulated inside *APOE3* iPSC-derived BMEC-like cells by confocal microscopy. (*n* = 3 independent differentiations for each condition). *: *p* < 0.05 by Student’s t-test. Data are reported as mean ± standard deviation. (**H**) Relative fluorescence units of Aβ40-Hylite488 accumulated inside *APOE3* iPSC-derived BMEC-like cells treated with DMSO or CsA. BMEC-like cells pretreated with DMSO or CsA were incubated with Aβ40-Hylite488. BMEC-like cells were washed and lysed. Fluorescence signals from lysates were quantified by plate reader. (*n* = 3 independent differentiations for each condition) *: *p* < 0.05 by Student’s t-test. Data are reported as mean ± standard deviation

Previous research has shown that APOE can bind to Aβ to form APOE-Aβ complexes [23, 74]. Both Aβ monomer and APOE-Aβ complexes can be cleared at the BBB [23, 38, 75–78] (Fig. 2A). BMEC-like cells are polarized, with LRP1 localized on the basolateral (brain) side and p-glycoprotein localized on the apical (blood) side [79–81]. Both LRP1 and p-glycoprotein have been implicated in brain-to-blood Aβ trafficking. Specifically, the APOE-Aβ complex binds to LRP1 and is then internalized into the cells. The complex can then be effluxed from BMEC-like cells by p-glycoprotein (Fig. 2A). Here, we assessed whether Aβ trafficking across the BMEC-like cell model had similar dependences on LRP1 and p-glycoprotein. We used immunocytochemistry to validate expression of LRP1 in *APOE3* iPSC-derived BMEC-like cells (Fig. 2B). To assess the effect of LRP1 in Aβ clearance, we developed an in vitro Aβ transcytosis assay to quantify the effect of APOE isoforms on brain-to-blood Aβ trafficking (Fig. 2C). *APOE3* iPSC-derived BMEC-like cells were seeded on a Transwell and allowed to form a confluent monolayer. Aβ was dosed into the basolateral chamber in the presence or absence of recombinant human APOE3 (rhAPOE3) and the LRP1 inhibitor RAP. RAP competes with APOE for LRP1 binding with an efficacy similar to anti-LRP1 antibodies [82, 83]. Using ELISA to quantify apical Aβ concentrations, we found that rhAPOE3 facilitated Aβ40 transcytosis and that inhibiting LRP1 with RAP reduced Aβ transcytosis (Fig. 2D). In addition, inhibiting LRP1 with RAP reduced but did not completely inhibit Aβ transcytosis, suggesting that alternative pathways are also likely involved in Aβ40 transport in BMEC-like cells. These data demonstrate that LRP1 is involved in basolateral-to-apical transport of Aβ in iPSC-derived BMEC like cells. We then used a p-glycoprotein activity assay to validate the function of p-glycoprotein, where Rh123 accumulation was increased in the presence of p-glycoprotein inhibitor cyclosporin A (CsA) as previously described [84] (Fig. 2E). To determine if p-glycoprotein plays a role in mediating Ab efflux in iPSC-derived BMEC-like cells, we incubated BMEC-like cells in medium containing fluorescently labeled Aβ40-Hylite488, with p-glycoprotein inhibitor CsA or DMSO negative control. Confocal microscopy was used to quantify the intracellular accumulation of Aβ40-Hylite488. CsA-treated BMEC-like cells had significantly higher intracellular accumulation of Aβ40-Hylite488 than DMSO control (Fig. 2F and G, Ext. Fig. 2). Cell lysates of BMEC-like cells treated with media containing Aβ40-Hylite488, with CsA or DMSO, were also collected for quantitative analysis by a fluorescence plate reader. Lysates of cells treated with CsA also contained significantly higher levels of Aβ40-Hylite488 than DMSO control, corroborating the microscopic evidence (Fig. 2H). Collectively, these data demonstrate that p-glycoprotein is capable of Aβ efflux in the iPSC-derived BMEC-like cell model.

### APOE isoforms affect amyloid clearance in iPSC-derived BMEC-like cells

**Fig. 3:**
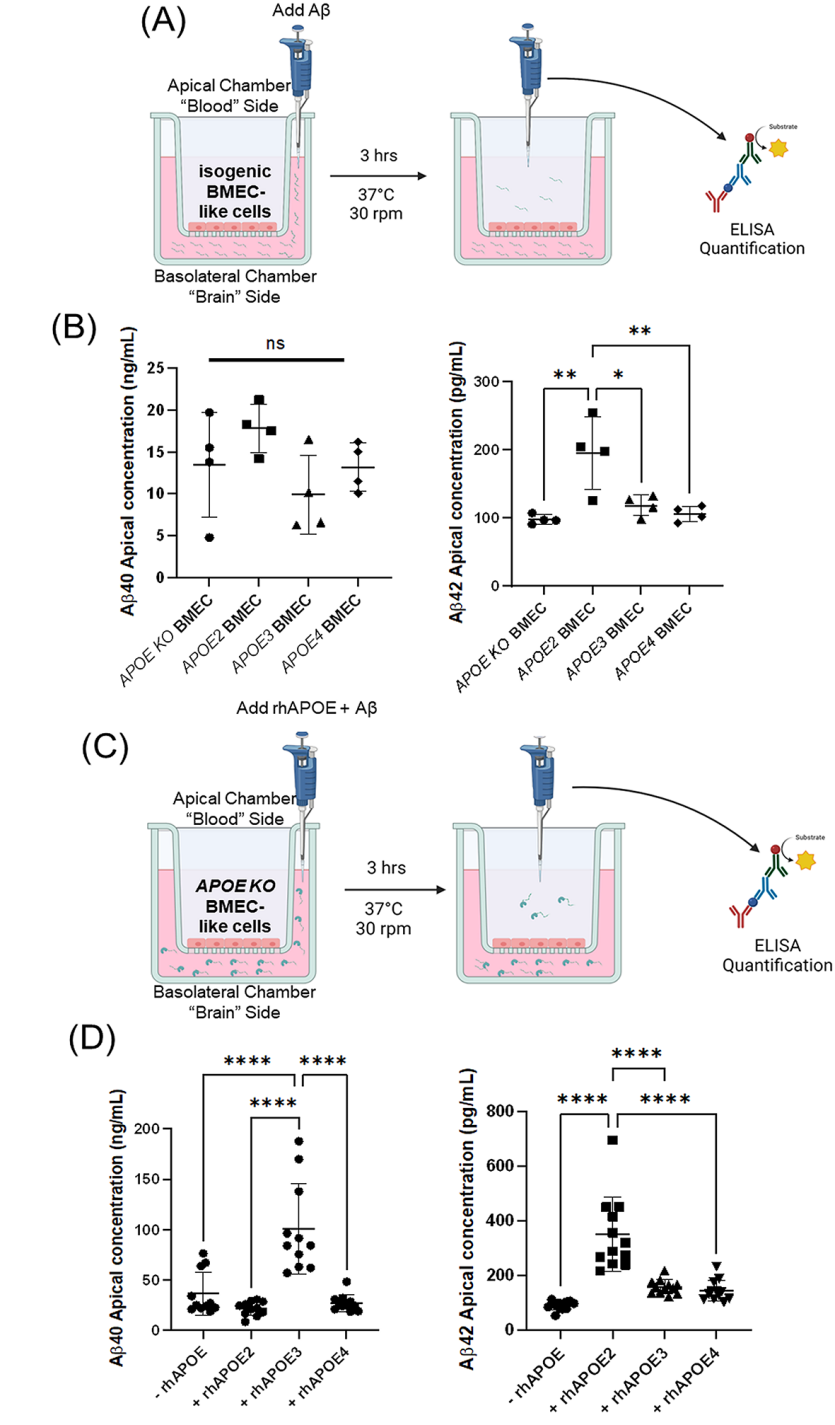
APOE isoforms alter the level of amyloid clearance in iPSC-derived BMEC-like cells. (**A**) Schematic showing experimental design analyzing the effect of different *APOE* genotypes on BMEC-like cells trafficking of Aβ. Isogenic BMEC-like cells with different *APOE* genotypes were seeded on a Transwell. 500nM Aβ40 or Aβ42 was dosed in the basolateral chamber. After a 3-hour incubation, Aβ concentrations in the apical chamber were quantified by ELISA. (**B**) Differential transport of Aβ40 or Aβ42 by isogenic BMEC-like cells to the apical chamber after 3 h of incubation quantified by ELISA. (*n* = 4 independent differentiations for each condition. Mean of three biological replicates in each differentiation is plotted.) *: *p* < 0.05, **: *p* < 0.01 in one-way ANOVA followed by Tukey’s test. Data are reported as mean ± standard deviation. (**C**) Schematic showing experimental design analyzing the effect of different APOE protein isoforms on BMEC-like cells trafficking of Aβ. *APOE KO* BMEC-like cells were seeded on a Transwell. 500nM Aβ40 or Aβ42 along with different isoforms of 500nM recombinant human APOE (rhAPOE) was dosed in the basolateral chamber. After a 3-hour incubation, Aβ40 or Aβ42 concentrations in the apical chamber were quantified by ELISA. (**D**) Differential transport of Aβ40 or Aβ42 by *APOE KO* BMEC-like cells with different recombinant APOE protein isoforms was measured in the apical chamber after 3 h of incubation quantified by ELISA. (*n* = 12 independent differentiations for each condition. Mean of three biological replicates in each differentiation is plotted.) ****: *p* < 0.0001 in one-way ANOVA followed by Tukey’s test. Data are reported as mean ± standard deviation

Using the in vitro Aβ transcytosis assay described above, the roles of different *APOE* genotypes on Aβ transport were quantitatively compared. Since Aβ monomer has been reported to be a ligand for LRP1-mediated transcytosis [23, 85–87], the two most common Aβ monomers, Aβ40 and Aβ42, were dosed into the basolateral chamber. After incubation, media in the apical chamber was collected. We then quantified the concentration of Aβ in the collected media using ELISA (Fig. 3A). While BMEC-like cells having different *APOE* genotypes transported Aβ40 at a similar rate, *APOE2* BMEC-like cells cleared more Aβ42 than isogenic BMEC-like cells from other *APOE* genotypes (Fig. 3B). Comparing Aβ40 and Aβ42 trafficking, we also noticed approximately two orders of magnitude greater Aβ40 clearance than Aβ42 (Fig. 3B and D, Ext. Fig. 3A). This may be a result of Aβ42 being more prone to aggregation and deposition compared to Aβ40 [88, 89], thus contributing to its reduced apparent clearance.

We next assessed the effect of APOE protein isoform on Aβ trafficking. To eliminate any background from endogenously produced APOE protein, *APOE KO* BMEC-like cells were seeded on the Transwell and allowed to form a confluent monolayer. Since the BMEC-like cells expressed very little (< 1.5 ng/mL, < 44 pM) APOE protein, we added 500 nM recombinant human APOE (rhAPOE), a physiological concentration [90, 91], to the basolateral chamber in media also containing Aβ40 or Aβ42, and ELISA was used to quantify Aβ concentrations in the apical chamber after incubation (Fig. 3C). We found that rhAPOE3 significantly increased the trafficking of Aβ40, and that supplementation of rhAPOE2 significantly increased the trafficking of Aβ42 (Fig. 3D). These data also suggest that rhAPOE4 does not provide any increase in Aβ40 or Aβ42 clearance compared to the no APOE control condition (Fig. 3D). Together, these data suggest that APOE2 mediated enhanced clearance of both free Aβ42 (Fig. 3B) and APOE-Aβ42 (Fig. 3D) complexes, while APOE3 enhanced clearance of APOE-Aβ40 complexes. Performing the transcytosis assay on *APOE KO* BMEC-like cells at 4 °C and 37 °C, we observed minimal transcytosis of Aβ40 at 4 °C, indicating that Aβ40 trafficking in this assay is largely transcellular (Ext. Figure 3B). These results are consistent with human clinical data where *APOE4* carriers exhibit significantly higher amyloid plaque deposition and *APOE2* carriers exhibit significantly lower amyloid deposition than AD patients with *APOE3/APOE3* genotypes [19, 92].

### iPSC-derived brain pericyte-like cells of different *APOE* genotypes have similar pericyte marker expression and APOE protein secretion profiles

**Fig. 4:**
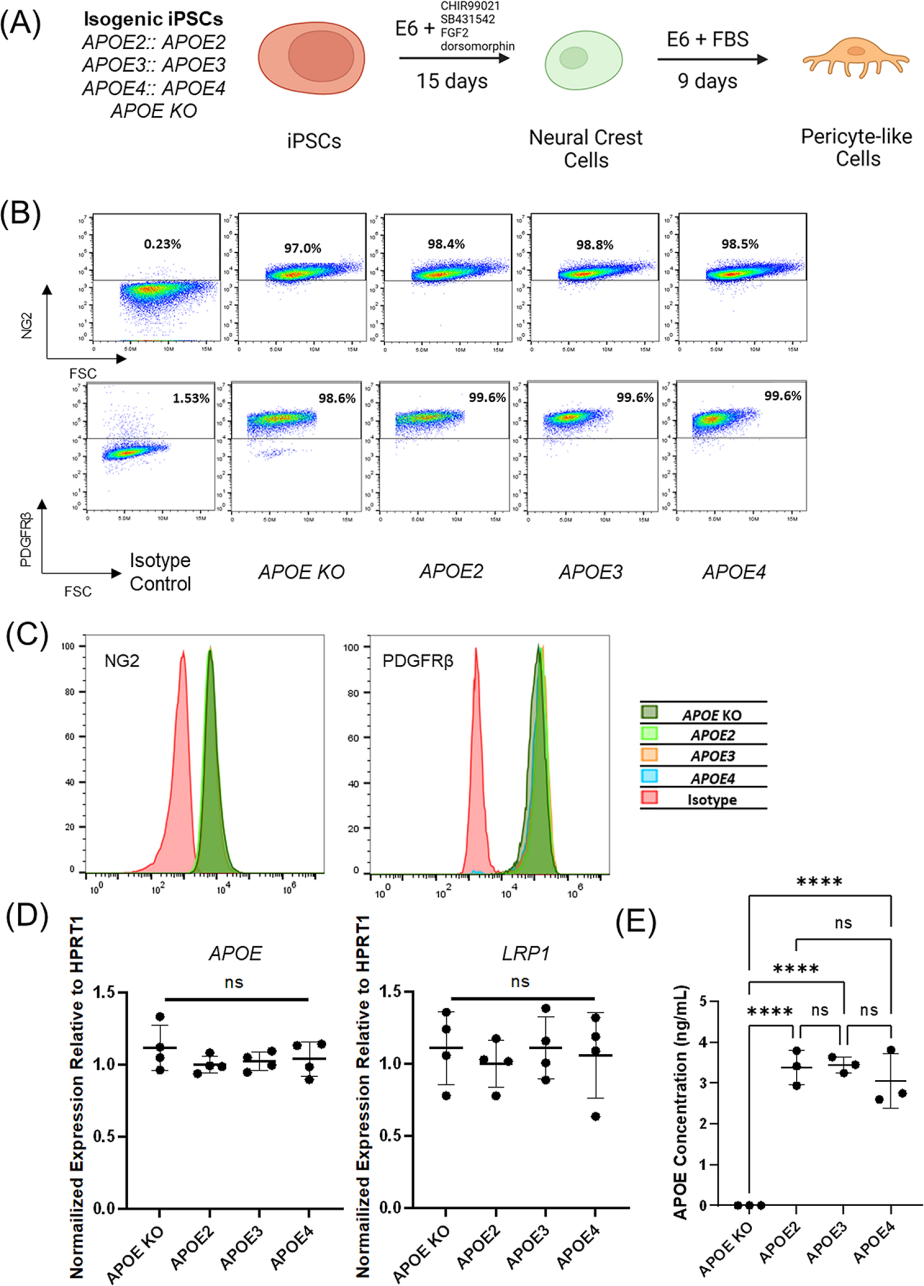
iPSC-derived brain pericyte-like cells with different *APOE* genotypes possess similar properties. (**A**) Isogenic iPSCs carrying different *APOE* genotypes were differentiated to brain pericyte-like cells via a neural crest cell progenitor as previously described [67]. (**B**) Flow cytometry analysis of NG2 and PDGFRβ expression in Day 24 isogenic pericyte-like cells. FSC: forward scatter. Gating is according to isotype control performed on a pooled population of Day 24 isogenic pericyte-like cells with different *APOE* genotypes. (**C**) Histograms of flow cytometry analysis in (**B**). Samples shown in histograms are normalized to mode. (**D**) RT-qPCR analysis of *APOE* and *LRP1* expression in Day 24 isogenic pericyte-like cells (*n* = 4 wells of independent differentiations for each condition). Bars indicate mean values. Data are normalized to the *APOE3* condition. ns: p-value > 0.05 in one-way ANOVA analysis. Data are reported as mean ± standard deviation. (**E**) Concentration of APOE protein in cell culture media conditioned by isogenic pericyte-like cells for 24 h. APOE concentration was quantified by ELISA. (*n* = 3 wells of independent differentiations for each condition). ****: p-value < 0.0001 in one-way ANOVA analysis followed by Tukey’s test. Data are reported as mean ± standard deviation

To determine how *APOE* genotype affects pericyte interactions with Aβ, established protocols were used to differentiate the isogenic, homozygous *APOE* iPSC lines into pericyte-like cells [67, 70] (Fig. 4A). After differentiation, pericyte-like cells derived from iPSCs with different *APOE* genotypes all expressed similar levels of pericyte markers NG2 and PDGFRβ as assessed flow cytometry (Fig. 4B and C). Since brain pericytes are one of the CNS cell types that secrete APOE [83], we quantified the expression level of *APOE* by RT-qPCR, and found that pericytes with different *APOE* genotypes expressed similar levels of *APOE* (Fig. 4D). We also quantified the concentrations of APOE protein secreted by pericyte-like cells by ELISA, and found no significant differences in total APOE protein among the *APOE2*, *APOE3*, and *APOE4* pericytes (Fig. 4E). We also found no significant differences in *LRP1* transcript expression between pericyte-like cells having different *APOE* alleles (Fig. 4D). These data suggest that the different *APOE* genotypes do not significantly affect canonical pericyte marker expression or *APOE* expression and APOE secretion levels.

### APOE isoforms affect amyloid deposition and internalization in iPSC-derived pericyte-like cells

**Fig. 5:**
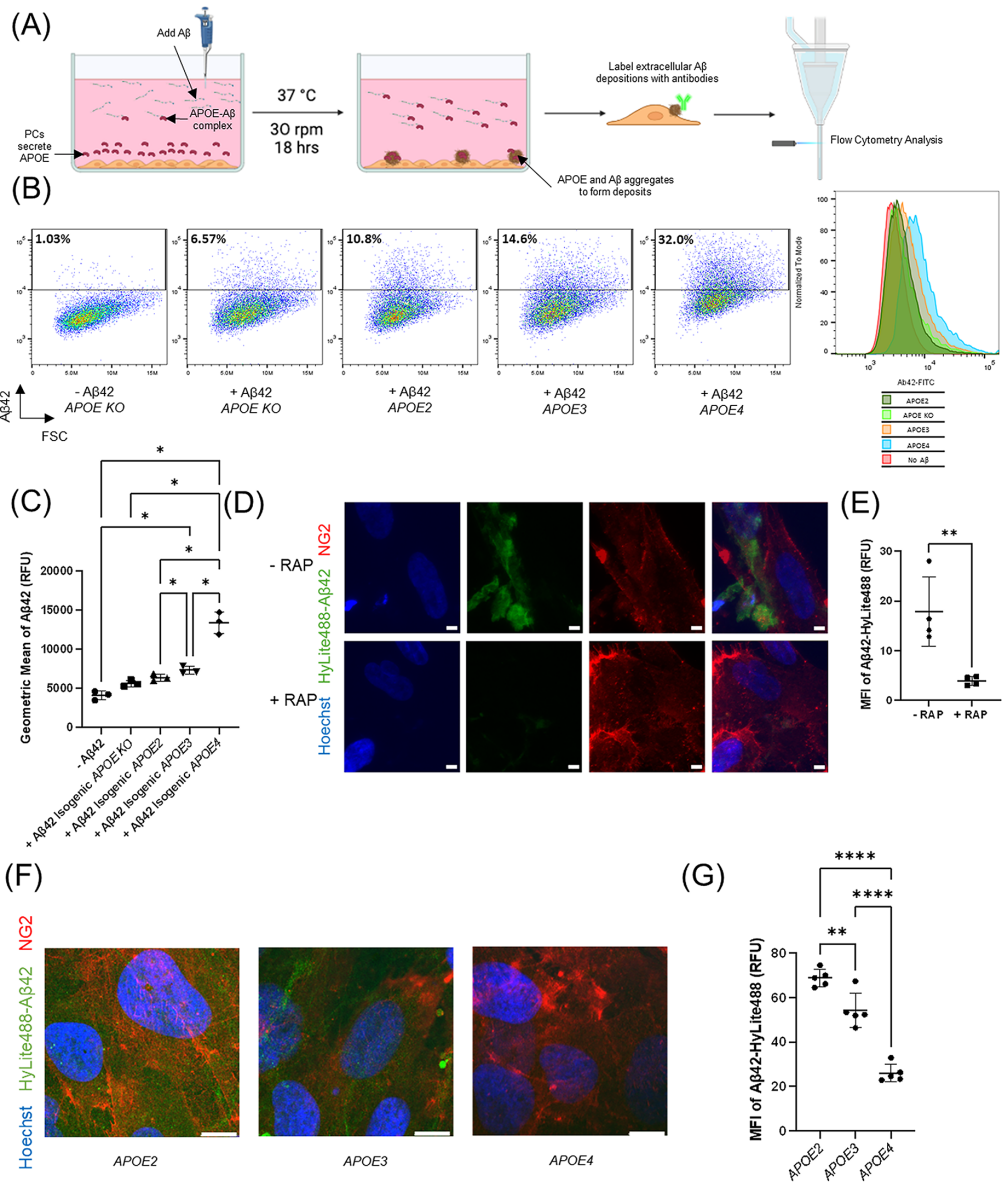
Isogenic iPSC-derived pericyte-like cells demonstrate differential Aβ42 deposition and internalization. (**A**) Schematic showing the experimental design analyzing the effect of different APOE isoforms on extracellular Aβ42 deposition. 500nM Aβ42 was incubated with pericyte-like cells for 18 h to allow deposition of extracellular Aβ aggregates. Pericyte-like cells were then singularized, washed and labeled with anti-Aβ-FITC antibody for flow cytometry quantification of cell surface-associated Aβ42. (**B**) Flow cytometry analysis of extracellular deposition of Aβ42 by isogenic pericyte-like cells after 18 h of incubation. FSC: forward scatter. Histogram is normalized to mode. (**C**) Quantification of level of Aβ42 deposition on pericytes by flow cytometry (*n* = 3 wells of biological replicates per condition). *: *p* < 0.05 in one-way ANOVA analysis followed by Tukey’s test. Data are reported as mean ± standard deviation. (**D**) Representative confocal microscopy images showing accumulation of intracellular Aβ42-Hylite488 with or without LRP1 inhibition by RAP in *APOE3* pericyte-like cells. Confocal microscopy images were taken at the z-plane where DAPI nucleus staining was visible to distinguish intracellular Aβ42-Hylite488. Images were then captured for the Aβ42-Hylite488 channel on the same z-plane. The last column contains the merged images. Scale bars: 10 μm. (**E**) Quantification of mean fluorescence intensity (MFI) of Aβ42-Hylite488 accumulated inside *APOE3* pericyte-like cells by confocal microscopy. (*n* = 4 wells of biological replicates per condition) **: *p* < 0.01 in Student’s t-test. Data are reported as mean ± standard deviation. (**F**) Maximum intensity projection of confocal microscopy images showing accumulation of intracellular Aβ42-Hylite488 by isogenic pericyte-like cells. Confocal microscopy images were taken at 5 μm z-slice intervals. All z-slices with Hoechst 33342 nuclear staining were aggregated for maximum intensity projection analysis. Scale bars: 10 μm. (**G**) Quantification of mean fluorescence intensity (MFI) in maximum intensity projection confocal microscopy images of Aβ42-Hylite488 accumulated inside iPSC-derived pericytes by confocal microscopy. (*n* = 5 slides of pericyte-like cells treated independently by Aβ42-Hylite488 per genotype). **:*p* < 0.01, ****:*p* < 0.0001 in one-way ANOVA followed by Tukey’s test

Patients with AD commonly have amyloid deposits associated with their brain vasculature, contributing to a pathological condition known as cerebral amyloid angiopathy (CAA) [93]. Recently, a role of pericytes in CAA has been reported [94]. We quantified deposition of Aβ42, the Aβ isoform more prone to aggregation, for pericyte-like cells expressing different APOE isoforms. We incubated pericyte-like cells differentiated from different *APOE* iPSCs with Aβ42 for 18 h. The pericyte-like cells were then singularized, washed and fixed, but not permeabilized, for cell surface immunolabeling with an anti-Aβ antibody. Flow cytometry was subsequently used to quantify extracellular deposits of Aβ42 (Fig. 5A). The *APOE KO* pericyte-like cells exhibited the least extracellular deposition of Aβ42 (Fig. 5B and C), indicating that APOE expression, regardless of isoform, exacerbates Aβ42 aggregation and deposition. A comparison of isogenic *APOE2*, *APOE3* and *APOE4* pericyte-like cells revealed that *APOE4* pericyte-like cells had the highest level of extracellular Aβ42 deposition while *APOE2* pericyte-like cells had the least amount of Aβ42 deposition (Fig. 5B and C). This is consistent with clinical data that in patients with CAA, *APOE4* carriers consistently demonstrate elevated perivascular amyloid in the brain [20, 59]. Since it has been reported that pericytes utilize LRP1 to internalize APOE-Aβ complexes [22, 76], we probed whether the iPSC-derived pericyte-like cells also utilize LRP1 to internalize Aβ42. Using confocal microscopy, we found that iPSC-derived *APOE3* pericyte-like cells internalized fluorescently labeled Aβ42-HyLite488 (Fig. 5D). However, when pericyte-like cells were treated with the LRP1 inhibitor RAP, internalization was diminished (Fig. 5D and E). In the same way, we then examined the impact of *APOE* genotype on pericyte-like cell internalization of Aβ42. *APOE4* pericyte-like cells internalized less Aβ42 than *APOE3* pericyte-like cells, while *APOE2* pericyte-like cells internalized the most Aβ42 (Fig. 5F and G). Overall, our data suggests that *APOE4* pericyte-like cells have higher levels of extracellular Aβ42 deposition but lower levels of internalization while *APOE2* pericyte-like cells have less extracellular deposition and more internalization of Aβ42.

## Discussion

AD is a progressive neurodegenerative disorder that leads to memory loss, cognitive decline, and eventual loss of ability to perform everyday activities [95]. Recently, many genetic risk factors have been identified for AD, including mutations in *APP*, *PSEN1*, *PSEN2* for familial AD [96], and mutations in *APOE*, *CLU*, *PICALM* for sporadic AD [97, 98]. Among these genetic risk factors, *APOE* has proven to be the strongest risk factor [7] with *APOE4* being detrimental and *APOE2* being protective. While neurons, astrocytes and microglia have been investigated as cellular targets for the differential effects of the *APOE* allele [99–102], the vascular contribution has more recently been a target of study given its direct role in Aβ trafficking and clearance. Clinical studies have linked *APOE4* with elevated leakage at the BBB, reduced pericyte coverage, and increased vascular amyloid deposition [21, 41, 54, 94, 103] compared to *APOE3* patient controls. These data have sparked interest in clarifying the role of different *APOE* isoforms at the BBB, given that BBB is thought to be the main site of clearance of Aβ [37]. One lingering question that is unanswered by clinical studies is whether the enhanced BBB dysfunction in *APOE4* carriers is a result of intrinsic deficiencies in BMECs or pericytes, or a result of chronic exposure to *APOE4*-producing astrocytes, enhanced neuroinflammation, or other pathologies associated with *APOE4* carriers [2, 4].

Here, by employing in vitro human iPSC-derived BBB models, we isolated two individual BBB cell types from chronic influences to better understand the roles of different *APOE* isoforms in BMEC-like cells and pericyte-like cells. By using isogenic iPSC lines with different *APOE* genotypes, we also minimized the effect of background genetic variation to further target the specific effects elicited by *APOE* isoforms. Overall, our work illustrated the multifaceted role that different APOE isoforms could play at the BBB. While different APOE isoforms do not directly affect standard properties of BMEC-like cells and pericyte-like cells, we observed differential Aβ clearance and deposition profiles associated with APOE isoforms. Our results collectively predict that *APOE4* would lead to more Aβ load in the CNS, while *APOE2* would reduce the Aβ load compared to the most common *APOE3* isoform. Furthermore, our results indicate that in BMEC-like cells, *APOE* isoforms do not influence intrinsic BMEC differentiation, and lead to similar BBB gene expression of efflux transporters, glucose transporter, and tight junction components in addition to indistinguishable paracellular barriers. Similarly, in pericyte-like cells, *APOE* isoforms do not affect pericyte differentiation, and do not influence the level of APOE protein secretion. These results highlight that simply possessing different *APOE* alleles does not directly affect BMEC-like cell and pericyte-like cell differentiation and function. These results are consistent with the premise that elevated BBB damage observed clinically in *APOE4* carriers, is at least in part the result of chronic pathological exposure from other *APOE4* CNS cell types such as neurons, astrocytes or microglia. The observed similarities in BMEC-like cell properties amongst isogenic lines in this study are in contrast with the differences observed in BMEC-like cells differentiated from iPSC lines derived from different AD patients, where differences in genetic background could potentially confound results [104].

We next validated the usage of the BMEC-like cells as an in vitro BBB model for Aβ trafficking studies by showing that the LRP1 and p-glycoprotein pathways are expressed and functional in Aβ trafficking. We then evaluated isogenic BMEC-like cells for differential Aβ trafficking effects associated with *APOE* allele expression. We showed that *APOE2* BMEC-like cells exhibited greater transcytosis of monomeric Aβ42 than *APOE3* and *APOE4* BMEC-like cells. For the transcytosis of APOE-Aβ complexes, APOE4 protein resulted in the lowest combined clearance of Aβ40 and Aβ42, while APOE3 protein increased the transport rate of Aβ40 and APOE2 protein increased the transport rate of Aβ42. These collective data are consistent with the putative biochemical mechanisms that drive altered Aβ trafficking by different APOE isoforms. For instance, monomeric Aβ, and APOE-Aβ complexes can be internalized through cell surface receptor LRP1, which has been suggested as the main brain-side Aβ clearance pathway utilized by brain endothelial cells [22, 37, 72, 75, 83]. Since monomeric Aβ has been reported to oligomerize into insoluble fibrils in the presence of APOE protein in a APOE4 > APOE3 > APOE2 fashion [7, 23], and oligomerized Aβ are less likely to be cleared by LRP1- based receptor-mediated transcytosis [76, 85, 105], differential Aβ oligomerization levels caused by different APOE isoforms could in part explain our observations. Our data also agree with clinical observations where Aβ brain depositions in AD patients are ranked in a *APOE4* > *APOE3* > *APOE2* fashion, suggesting differential clearance of Aβ with different *APOE* isoforms [23, 106–108]. One caveat of the iPSC-derived BMEC-like cells used in this manuscript is the existence of a mixed endothelial-epithelial phenotype [109, 110] within the homogenous cell population. While other protocols generate iPSC-derived BBB-like cells without the epithelial character, the resultant cells lack tight enough paracellular barriers [109, 111, 112] to discern differences in transcellular Aβ passage. By contrast, the iPSC-derived BMEC-like cells used here possess requisite tightness along with functional LRP1 and p-glycoprotein mediated Aβ transport pathways, indicating their relevance for Aβ transport modeling.

Vascular Aβ deposition has been shown to be related to pericytes in AD and cerebral amyloid angiopathy (CAA), and vascular Aβ deposition is dependent on APOE isoform, with an *APOE4* > *APOE3* > *APOE2* trend [94, 113, 114]. Thus, we also assessed the role of *APOE* alleles in pericyte-like cells in contributing to Aβ deposition and uptake. For extracellular Aβ42 deposition on pericyte-like cells, a pattern of *APOE4* > *APOE3* > *APOE2* was observed. For Aβ42 internalization, we found that *APOE4* pericyte-like cells exhibited the least Aβ42 uptake compared to *APOE2* and *APOE3* pericyte-like cells. Since Aβ can be degraded by intracellular lysosome-dependent pathways [26–29], a reduced internalization could also contribute to increased amyloid load in the brain. These combined data suggest a potential detrimental role for *APOE4* in increasing extracellular Aβ42 deposition while also decreasing Aβ42 internalization for potential degradation. In contrast, *APOE2* improves the Aβ42 deposition profile. Our results also confirm a previously reported pathway that pericytes can internalize Aβ via LRP1 receptors [83], while providing additional insights into the effect of pericytes *APOE* isoforms on Aβ42 internalization. Differential Aβ42 association with pericyte-like cells was observed despite indistinguishable APOE secretion, which could be a result of the differential biochemical association of Aβ42 with the different APOE isoforms [23]. Previous reports have suggested that isogenic *APOE4* iPSC-derived pericyte-like cells secrete more APOE compared to *APOE3* controls^94^. This difference with our work could potentially be attributed to the use of different pericyte differentiation protocols. In this work, pericyte-like cells were differentiated through neural crest stem cells [67], analogous to the developmental profile of forebrain pericytes [115–118], while pericyte-like cells in the contrasting work were derived from a mesodermal lineage [94, 119].

Finally, this work highlights the potential of using isogenic iPSC lines for modeling AD effects on the BBB. We demonstrated that isogenic iPSC-derived in vitro BBB models successfully captured aspects of AD-related amyloid pathologies by illustrating the effects of different APOE isoforms on Aβ trafficking and deposition. These in vitro BBB models could prove helpful in studying AD, and in the future, they could be used to isolate cell type-specific effects and help identify mechanisms by which the disease alters the BBB. For instance, previous studies using different stem cell-based in vitro BBB models have demonstrated applications in the modeling of BBB permeability after exposure to AD neural stem cell-derived neurons [120], and amyloid deposition modeling using vascular assembloids with pluripotent stem cell-derived AD mural cells [94]. Specifically, through the use of gene-edited isogenic iPSC lines carrying pathogenic mutations, researchers can explore potential disease influencing impacts with minimal influence from the genetic background [121]. Such efforts have been applied to study the effect of pathogenic mutations in Down syndrome [122], Parkinson’s Disease [123] and AD [94, 124, 125]. We envision that similar strategies could illustrate the role of other pathogenic mutations at the BBB in neurological diseases such as AD.

## Conclusions

iPSC-derived BBB models can capture aspects of amyloid pathologies at the BBB. Different *APOE* genotypes did not directly affect general BMEC-like cell or pericyte-like cell properties in the iPSC-derived cells. However, recombinant APOE4 (BMEC-like cells) and *APOE4* genotype (pericyte-like cells) exacerbated amyloid clearance and deposition at the BBB. In addition, *APOE2* demonstrated a protective role against amyloid pathologies using the models. These results are consistent with clinical observations on the effect of different *APOE* genotypes on CNS amyloid load.

## Electronic supplementary material

Below is the link to the electronic supplementary material.

Supplementary Material 1

## Data Availability

All data supporting the findings of this study are available within the paper and its Supplementary Information.

## Abbreviations

BBB: Blood-brain barrier
BMEC-like cells: Brain microvascular endothelial cell-like cells
CNS: Central nervous system
NVU: Neurovascular unit
AD: Alzheimer’s Disease
hPSC: Human pluripotent stem cell
iPSC: Induced pluripotent stem cell
CAA: Cerebral amyloid angiopathy
Aβ: Amyloid beta
Aβ40: Amyloid beta 1–40
Aβ42: Amyloid beta 1–42
APOE: Apolipoprotein E

## References

[1] Villabona-Rueda A, Erice C, Pardo CA, Stins MF. The Evolving Concept of the blood brain barrier (BBB): from a single static barrier to a heterogeneous and dynamic Relay Center. Front Cell Neurosci. 2019;13:405. 31616251 10.3389/fncel.2019.00405 PMC6763697

[2] Yu X, Ji C, Shao A. Neurovascular unit dysfunction and neurodegenerative disorders. Front Neurosci. 2020;14:334. 32410936 10.3389/fnins.2020.00334 PMC7201055

[3] Daneman R, Prat A. The blood-brain barrier. Cold Spring Harb Perspect Biol. 2015;7:a020412. 25561720 10.1101/cshperspect.a020412 PMC4292164

[4] Cai Z, et al. Role of blood-brain barrier in Alzheimer’s disease. J Alzheimers Dis. 2018;63:1223–34. 29782323 10.3233/JAD-180098

[5] Al-Bachari S, Naish JH, Parker GJM, Emsley HCA, Parkes LM. Blood-brain barrier leakage is increased in Parkinson’s Disease. Front Physiol. 2020;11:593026. 33414722 10.3389/fphys.2020.593026 PMC7784911

[6] Li X, et al. Global, regional, and national burden of Alzheimer’s disease and other dementias, 1990–2019. Front Aging Neurosci. 2022;14:937486. 36299608 10.3389/fnagi.2022.937486 PMC9588915

[7] Raulin A-C, et al. ApoE in Alzheimer’s disease: pathophysiology and therapeutic strategies. Mol Neurodegener. 2022;17:72. 36348357 10.1186/s13024-022-00574-4 PMC9644639

[8] Guo T, et al. Molecular and cellular mechanisms underlying the pathogenesis of Alzheimer’s disease. Mol Neurodegener. 2020;15:40. 32677986 10.1186/s13024-020-00391-7 PMC7364557

[9] DeTure MA, Dickson DW. The neuropathological diagnosis of Alzheimer’s disease. Mol Neurodegener. 2019;14:32. 31375134 10.1186/s13024-019-0333-5 PMC6679484

[10] Serrano-Pozo A, Frosch MP, Masliah E, Hyman BT. Neuropathological alterations in Alzheimer disease. Cold Spring Harb Perspect Med. 2011;1:a006189. 22229116 10.1101/cshperspect.a006189 PMC3234452

[11] Raulin A-C, Martens YA, Bu G. Lipoproteins in the Central Nervous System: from Biology to Pathobiology. Annu Rev Biochem. 2022;91:731–59. 35303786 10.1146/annurev-biochem-032620-104801 PMC9634960

[12] Lambert JC, et al. Meta-analysis of 74,046 individuals identifies 11 new susceptibility loci for Alzheimer’s disease. Nat Genet. 2013;45:1452–8. 24162737 10.1038/ng.2802 PMC3896259

[13] Huang Y, Mahley RW. Apolipoprotein E: structure and function in lipid metabolism, neurobiology, and Alzheimer’s diseases. Neurobiol Dis. 2014;72 Pt A:3–12. 10.1016/j.nbd.2014.08.025 PMC4253862 25173806

[14] Getz GS, Reardon CA. 2009:Suppl. S156–61. 10.1194/jlr.R800058-JLR200 PMC2674757 19018038

[15] Liao F, Yoon H, Kim J. Apolipoprotein E metabolism and functions in brain and its role in Alzheimer’s disease. Curr Opin Lipidol. 2017;28:60–7. 27922847 10.1097/MOL.0000000000000383 PMC5213812

[16] Johnson LA. APOE at the BBB: Pericyte-derived apolipoprotein E4 diminishes endothelial cell barrier function. Arteriosclerosis, thrombosis, and vascular biology. 2020;40:14–16. 10.1161/ATVBAHA.119.313627 PMC7001796 31869265

[17] Abondio P et al. The genetic variability of APOE in different human populations and its implications for longevity. Genes. 2019;10. 10.3390/genes10030222 PMC6471373 30884759

[18] Husain MA, Laurent B, Plourde M. APOE and Alzheimer’s Disease: from lipid transport to physiopathology and therapeutics. Front Neurosci. 2021;15:630502. 33679311 10.3389/fnins.2021.630502 PMC7925634

[19] Li Z, Shue F, Zhao N, Shinohara M, Bu G. APOE2: protective mechanism and therapeutic implications for Alzheimer’s disease. Mol Neurodegener. 2020;15:63. 33148290 10.1186/s13024-020-00413-4 PMC7640652

[20] Liu C-C, et al. ApoE4 accelerates early seeding of amyloid Pathology. Neuron. 2017;96:1024–e10323. 29216449 10.1016/j.neuron.2017.11.013 PMC5948105

[21] Montagne A, et al. APOE4 leads to blood-brain barrier dysfunction predicting cognitive decline. Nature. 2020;581:71–6. 32376954 10.1038/s41586-020-2247-3 PMC7250000

[22] Tachibana M, et al. APOE4-mediated amyloid-β pathology depends on its neuronal receptor LRP1. J Clin Invest. 2019. 10.1172/JCI124853. 10.1172/JCI124853 PMC6391135 30741718

[23] Kanekiyo T, Xu H, Bu G. ApoE and Aβ in Alzheimer’s disease: accidental encounters or partners? Neuron. 2014;81:740–54. 24559670 10.1016/j.neuron.2014.01.045 PMC3983361

[24] Baek MS, et al. Effect of APOE ε4 genotype on amyloid-β and tau accumulation in Alzheimer’s disease. Alzheimers Res Ther. 2020;12:1–12. 10.1186/s13195-020-00710-6 PMC7603688 33129364

[25] Insel PS, Hansson O. Mattsson-Carlgren, N. Association between Apolipoprotein E ε2 vs ε4, Age, and β-Amyloid in adults without cognitive impairment. JAMA Neurol. 2021;78:229–35. 33044487 10.1001/jamaneurol.2020.3780 PMC7551211

[26] Li J, et al. Differential regulation of amyloid-β endocytic trafficking and lysosomal degradation by apolipoprotein E isoforms. J Biol Chem. 2012;287:44593–601. 23132858 10.1074/jbc.M112.420224 PMC3531774

[27] Fote GM et al. Isoform-dependent lysosomal degradation and internalization of apolipoprotein E requires autophagy proteins. J Cell Sci. 2022;135. 10.1242/jcs.258687 PMC8917355 34982109

[28] Nuriel T, et al. The endosomal-lysosomal pathway is dysregulated by APOE4 expression in vivo. Front Neurosci. 2017;11:702. 29311783 10.3389/fnins.2017.00702 PMC5733017

[29] Lee H, et al. ApoE4-dependent lysosomal cholesterol accumulation impairs mitochondrial homeostasis and oxidative phosphorylation in human astrocytes. Cell Rep. 2023;42:113183. 37777962 10.1016/j.celrep.2023.113183

[30] Kloske CM, Wilcock DM. The important interface between apolipoprotein E and neuroinflammation in Alzheimer’s Disease. Front Immunol. 2020;11:754. 32425941 10.3389/fimmu.2020.00754 PMC7203730

[31] Parhizkar S, Holtzman DM. APOE mediated neuroinflammation and neurodegeneration in Alzheimer’s disease. Semin Immunol. 2022;59:101594. 35232622 10.1016/j.smim.2022.101594 PMC9411266

[32] Arnaud L, et al. APOE4 drives inflammation in human astrocytes via TAGLN3 repression and NF-κB activation. Cell Rep. 2022;40:111200. 35977506 10.1016/j.celrep.2022.111200

[33] Hasel P, Liddelow SA. Isoform-dependent APOE secretion modulates neuroinflammation. Nat Reviews Neurol. 2021;17:265–6. 10.1038/s41582-021-00483-y 33727705

[34] Wang D, et al. Relationship between Amyloid-β deposition and blood-brain barrier dysfunction in Alzheimer’s Disease. Front Cell Neurosci. 2021;15:695479. 34349624 10.3389/fncel.2021.695479 PMC8326917

[35] Bellaver B, et al. Blood-brain barrier integrity impacts the use of plasma amyloid-β as a proxy of brain amyloid-β pathology. Alzheimers Dement. 2023;19:3815–25. 36919582 10.1002/alz.13014 PMC10502181

[36] Deane R, et al. RAGE mediates amyloid-beta peptide transport across the blood-brain barrier and accumulation in brain. Nat Med. 2003;9:907–13. 12808450 10.1038/nm890

[37] Nelson AR, Sagare AP, Zlokovic BV. Chapter 9 - blood–brain barrier transport of Alzheimer’s amyloid β-Peptide. In: Wolfe MS, editor. Developing therapeutics for Alzheimer’s Disease. Boston: Academic. 2016:251–70. 10.1016/B978-0-12-802173-6.00009-5.

[38] Shibata M, et al. Clearance of Alzheimer’s amyloid-ss(1–40) peptide from brain by LDL receptor-related protein-1 at the blood-brain barrier. J Clin Invest. 2000;106:1489–99. 11120756 10.1172/JCI10498 PMC387254

[39] Leite M. Syndapin-2 mediated transcytosis of amyloid-β across the blood-brain barrier. Brain Commun. 2022;4:fcac039. 35233527 10.1093/braincomms/fcac039 PMC8882007

[40] Reas ET et al. Blood-brain barrier permeability is increased in early Alzheimer’s disease and correlates with brain microstructure. Alzheimers Dement. 2022;18.

[41] Sweeney MD, Sagare AP, Zlokovic BV. Blood-brain barrier breakdown in Alzheimer disease and other neurodegenerative disorders. Nat Rev Neurol. 2018;14:133–50. 29377008 10.1038/nrneurol.2017.188 PMC5829048

[42] Verma N, et al. Aβ efflux impairment and inflammation linked to cerebrovascular accumulation of amyloid-forming amylin secreted from pancreas. Commun Biol. 2023;6:2. 36596993 10.1038/s42003-022-04398-2 PMC9810597

[43] Mawuenyega KG, et al. Decreased clearance of CNS β-amyloid in Alzheimer’s disease. Science. 2010;330:1774. 21148344 10.1126/science.1197623 PMC3073454

[44] Sun Z, et al. Reduction in pericyte coverage leads to blood-brain barrier dysfunction via endothelial transcytosis following chronic cerebral hypoperfusion. Fluids Barriers CNS. 2021;18:21. 33952281 10.1186/s12987-021-00255-2 PMC8101037

[45] Uemura MT, Maki T, Ihara M, Lee VMY, Trojanowski JQ. Brain microvascular pericytes in Vascular Cognitive Impairment and Dementia. Front Aging Neurosci. 2020;12:80. 32317958 10.3389/fnagi.2020.00080 PMC7171590

[46] Rezai-Zadeh K, Gate D, Town T. CNS infiltration of peripheral immune cells: D-Day for neurodegenerative disease? J Neuroimmune Pharmacol. 2009;4:462–75. 19669892 10.1007/s11481-009-9166-2 PMC2773117

[47] Zang X, Chen S, Zhu J, Ma J, Zhai Y. The emerging role of Central and Peripheral Immune systems in neurodegenerative diseases. Front Aging Neurosci. 2022;14:872134. 35547626 10.3389/fnagi.2022.872134 PMC9082639

[48] Yamazaki Y, et al. Selective loss of cortical endothelial tight junction proteins during Alzheimer’s disease progression. Brain. 2019;142:1077–92. 30770921 10.1093/brain/awz011 PMC6439325

[49] Howe MD, McCullough LD, Urayama A. The role of basement membranes in cerebral amyloid Angiopathy. Front Physiol. 2020;11:601320. 33329053 10.3389/fphys.2020.601320 PMC7732667

[50] Perlmutter LS, Myers MA, Barrón E. Vascular basement membrane components and the lesions of Alzheimer’s disease: light and electron microscopic analyses. Microsc Res Tech. 1994;28:204–15. 8068983 10.1002/jemt.1070280305

[51] Zarow C, Barron E, Chui HC, Perlmutter LS. Vascular basement membrane pathology and Alzheimer’s disease. Ann N Y Acad Sci. 1997;826:147–60. 9329687 10.1111/j.1749-6632.1997.tb48467.x

[52] Bertram L, Tanzi RE. Genome-wide association studies in Alzheimer’s disease. Hum Mol Genet. 2009;18:R137–45. 19808789 10.1093/hmg/ddp406 PMC2758713

[53] Jackson RJ, et al. APOE4 derived from astrocytes leads to blood-brain barrier impairment. Brain. 2021. 10.1093/brain/awab478. 10.1093/brain/awab478 PMC9586546 34957486

[54] Barisano G et al. A multi-omics analysis of blood-brain barrier and synaptic dysfunction in APOE4 mice. J Exp Med. 2022;219. 10.1084/jem.20221137 PMC9435921 36040482

[55] Yamazaki Y, et al. ApoE (apolipoprotein E) in Brain Pericytes regulates endothelial function in an isoform-dependent manner by modulating basement membrane components. Arterioscler Thromb Vasc Biol. 2020;40:128–44. 31665905 10.1161/ATVBAHA.119.313169 PMC7007705

[56] Halliday MR, et al. Accelerated pericyte degeneration and blood-brain barrier breakdown in apolipoprotein E4 carriers with Alzheimer’s disease. J Cereb Blood Flow Metab. 2016;36:216–27. 25757756 10.1038/jcbfm.2015.44 PMC4758554

[57] Zhou X, et al. ApoE4-mediated blood-brain barrier damage in Alzheimer’s disease: Progress and prospects. Brain Res Bull. 2023;199:110670. 37224887 10.1016/j.brainresbull.2023.110670

[58] Gharbi-Meliani A, et al. The association of APOE ε4 with cognitive function over the adult life course and incidence of dementia: 20 years follow-up of the Whitehall II study. Alzheimers Res Ther. 2021;13:5. 33397450 10.1186/s13195-020-00740-0 PMC7784268

[59] Emrani S, Arain HA, DeMarshall C, Nuriel T. APOE4 is associated with cognitive and pathological heterogeneity in patients with Alzheimer’s disease: a systematic review. Alzheimers Res Ther. 2020;12:141. 33148345 10.1186/s13195-020-00712-4 PMC7643479

[60] Vemuri P, et al. Effect of apolipoprotein E on biomarkers of amyloid load and neuronal pathology in Alzheimer disease. Ann Neurol. 2010;67:308–16. 20373342 10.1002/ana.21953 PMC2886799

[61] Liu C-C, Liu C-C, Kanekiyo T, Xu H, Bu G. Apolipoprotein E and Alzheimer disease: risk, mechanisms and therapy. Nat Rev Neurol. 2013;9:106–18. 23296339 10.1038/nrneurol.2012.263 PMC3726719

[62] Liu C-C, et al. Peripheral apoE4 enhances Alzheimer’s pathology and impairs cognition by compromising cerebrovascular function. Nat Neurosci. 2022;25:1020–33. 35915180 10.1038/s41593-022-01127-0 PMC10009873

[63] Zenaro E, Piacentino G, Constantin G. The blood-brain barrier in Alzheimer’s disease. Neurobiol Dis. 2017;107:41–56. 27425887 10.1016/j.nbd.2016.07.007 PMC5600438

[64] Lippmann ES, et al. Derivation of blood-brain barrier endothelial cells from human pluripotent stem cells. Nat Biotechnol. 2012;30:783–91. 22729031 10.1038/nbt.2247 PMC3467331

[65] Lippmann ES, Al-Ahmad A, Azarin SM, Palecek SP, Shusta E. V. A retinoic acid-enhanced, multicellular human blood-brain barrier model derived from stem cell sources. Sci Rep. 2014;4:4160. 24561821 10.1038/srep04160 PMC3932448

[66] Stebbins MJ, et al. Activation of RARα, RARγ, or RXRα increases Barrier Tightness in Human Induced Pluripotent Stem cell-derived brain endothelial cells. Biotechnol J. 2018;13:1–12. 10.1002/biot.201700093 PMC5796863 28960887

[67] Stebbins MJ, et al. Human pluripotent stem cell-derived brain pericyte-like cells induce blood-brain barrier properties. Sci Adv. 2019;5:eaau7375. 30891496 10.1126/sciadv.aau7375 PMC6415958

[68] Stebbins MJ, et al. Differentiation and characterization of human pluripotent stem cell-derived brain microvascular endothelial cells. Methods. 2016;101:93–102. 26518252 10.1016/j.ymeth.2015.10.016 PMC4848177

[69] Stebbins MJ et al. Human pluripotent stem cell–derived brain pericyte–like cells induce blood-brain barrier properties. Sci Adv. 2019;5. 10.1126/sciadv.aau7375 PMC6415958 30891496

[70] Gastfriend BD, Stebbins MJ, Du F, Shusta EV, Palecek SP. Differentiation of Brain Pericyte-Like cells from human pluripotent stem cell-derived neural crest. Curr Protoc. 2021;1:e21. 33484491 10.1002/cpz1.21 PMC7839246

[71] Kim BJ et al. Modeling Group B Streptococcus and Blood-Brain Barrier Interaction by Using Induced Pluripotent Stem Cell-Derived Brain Endothelial Cells. mSphere. 2017;2:1–12. 10.1128/mSphere.00398-17 PMC5663983 29104935

[72] Nikolakopoulou AM et al. Endothelial LRP1 protects against neurodegeneration by blocking cyclophilin A. J Exp Med. 2021;218. 10.1084/jem.20202207 PMC7863706 33533918

[73] Yan SD, Bierhaus A, Nawroth PP, Stern DM. RAGE and Alzheimer’s disease: a progression factor for amyloid-beta-induced cellular perturbation? J Alzheimers Dis. 2009;16:833–43. 19387116 10.3233/JAD-2009-1030 PMC3726270

[74] Wisniewski T, Drummond E. APOE-amyloid interaction: therapeutic targets. Neurobiol Dis. 2020;138:104784. 32027932 10.1016/j.nbd.2020.104784 PMC7118587

[75] Storck SE, et al. Endothelial LRP1 transports amyloid-β(1–42) across the blood-brain barrier. J Clin Invest. 2016;126:123–36. 26619118 10.1172/JCI81108 PMC4701557

[76] Van Gool B, et al. LRP1 has a predominant role in production over clearance of Aβ in a mouse model of Alzheimer’s Disease. Mol Neurobiol. 2019;56:7234–45. 31004319 10.1007/s12035-019-1594-2 PMC6728278

[77] Martiskainen H, et al. Targeting ApoE4/ApoE receptor LRP1 in Alzheimer’s disease. Expert Opin Ther Targets. 2013;17:781–94. 23573918 10.1517/14728222.2013.789862

[78] Hultman K, Strickland S, Norris EH. The APOE ε4/ε4 genotype potentiates vascular fibrin(ogen) deposition in amyloid-laden vessels in the brains of Alzheimer’s disease patients. J Cereb Blood Flow Metab. 2013. 10.1038/jcbfm.2013.76. 10.1038/jcbfm.2013.76 PMC3734776 23652625

[79] Storck SE, et al. The concerted amyloid-beta clearance of LRP1 and ABCB1/P-gp across the blood-brain barrier is linked by PICALM. Brain Behav Immun. 2018;73:21–33. 30041013 10.1016/j.bbi.2018.07.017 PMC7748946

[80] Thiebaut F, et al. Cellular localization of the multidrug-resistance gene product P-glycoprotein in normal human tissues. Proc Natl Acad Sci U S A. 1987;84:7735–8. 2444983 10.1073/pnas.84.21.7735 PMC299375

[81] Fu D. Where is it and how does it get there - intracellular localization and traffic of P-glycoprotein. Front Oncol. 2013;3:321. 24416721 10.3389/fonc.2013.00321 PMC3874554

[82] Fagan AM, Bu G, Sun Y, Daugherty A, Holtzman DM. Apolipoprotein E-containing high density lipoprotein promotes neurite outgrowth and is a ligand for the low density lipoprotein receptor-related protein. J Biol Chem. 1996;271:30121–5. 8939961 10.1074/jbc.271.47.30121

[83] Ma Q, et al. Blood-brain barrier-associated pericytes internalize and clear aggregated amyloid-β42 by LRP1-dependent apolipoprotein E isoform-specific mechanism. Mol Neurodegener. 2018;13:57. 30340601 10.1186/s13024-018-0286-0 PMC6194676

[84] Twentyman PR, Rhodes T, Rayner S. A comparison of rhodamine 123 accumulation and efflux in cells with P-glycoprotein-mediated and MRP-associated multidrug resistance phenotypes. Eur J Cancer. 1994;30A:1360–9. 7999426 10.1016/0959-8049(94)90187-2

[85] Kanekiyo T, Bu G. The low-density lipoprotein receptor-related protein 1 and amyloid-β clearance in Alzheimer’s disease. Front Aging Neurosci. 2014;6:93. 24904407 10.3389/fnagi.2014.00093 PMC4033011

[86] Yamazaki Y, Zhao N, Caulfield TR, Liu C-C, Bu G. Apolipoprotein E and Alzheimer disease: pathobiology and targeting strategies. Nat Rev Neurol. 2019;15:501–18. 31367008 10.1038/s41582-019-0228-7 PMC7055192

[87] Robert J, et al. Cerebrovascular amyloid Angiopathy in bioengineered vessels is reduced by high-density lipoprotein particles enriched in apolipoprotein E. Mol Neurodegener. 2020;15:23. 32213187 10.1186/s13024-020-00366-8 PMC7093966

[88] Phillips JC. Why Aβ42 is much more toxic than Aβ40. ACS Chem Neurosci. 2019;10:2843–7. 31042351 10.1021/acschemneuro.9b00068

[89] Pauwels K, et al. Structural basis for increased toxicity of pathological Aβ42:Aβ40 ratios in alzheimer disease. J Biol Chem. 2012. 10.1074/jbc.M111.264473. 10.1074/jbc.M111.264473 PMC3285338 22157754

[90] Wahrle SE, et al. Apolipoprotein E levels in cerebrospinal fluid and the effects of ABCA1 polymorphisms. Mol Neurodegener. 2007;2:7. 17430597 10.1186/1750-1326-2-7 PMC1857699

[91] Rezeli M, et al. Quantification of total apolipoprotein E and its specific isoforms in cerebrospinal fluid and blood in Alzheimer’s disease and other neurodegenerative diseases. EuPA Open Proteom. 2015;8:137–43.

[92] Drzezga A, et al. Effect of APOE genotype on amyloid plaque load and gray matter volume in Alzheimer disease. Neurology. 2009;72:1487–94. 19339712 10.1212/WNL.0b013e3181a2e8d0

[93] Ringman JM, et al. Clinical predictors of severe cerebral amyloid angiopathy and influence of APOE genotype in persons with pathologically verified Alzheimer disease. JAMA Neurol. 2014;71:878–83. 24797962 10.1001/jamaneurol.2014.681 PMC4101018

[94] Blanchard JW, et al. Reconstruction of the human blood-brain barrier in vitro reveals a pathogenic mechanism of APOE4 in pericytes. Nat Med. 2020;26:952–63. 32514169 10.1038/s41591-020-0886-4 PMC7704032

[95] 2023 Alzheimer’s disease facts and figures. Alzheimers. Dement. 2023;19:1598–1695. 10.1002/alz.13016 36918389

[96] Reitz C, Pericak-Vance MA, Foroud T, Mayeux R. A global view of the genetic basis of Alzheimer disease. Nat Rev Neurol. 2023;19:261–77. 37024647 10.1038/s41582-023-00789-z PMC10686263

[97] Harold D, et al. Genome-wide association study identifies variants at CLU and PICALM associated with Alzheimer’s disease. Nat Genet. 2009;41:1088–93. 19734902 10.1038/ng.440 PMC2845877

[98] Bellenguez C, et al. New insights into the genetic etiology of Alzheimer’s disease and related dementias. Nat Genet. 2022;54:412–36. 35379992 10.1038/s41588-022-01024-z PMC9005347

[99] Huang YWA, Zhou B, Wernig M, Südhof TC. ApoE2, ApoE3, and ApoE4 differentially stimulate APP transcription and Aβ secretion. Cell. 2017;168:427–e44121. 28111074 10.1016/j.cell.2016.12.044 PMC5310835

[100] Wang C, et al. Gain of toxic apolipoprotein E4 effects in human iPSC-derived neurons is ameliorated by a small-molecule structure corrector. Nat Med. 2018;24:647–57. 29632371 10.1038/s41591-018-0004-z PMC5948154

[101] Lin Y-T, et al. APOE4 causes widespread molecular and cellular alterations associated with Alzheimer’s disease phenotypes in human iPSC-derived brain cell types. Neuron. 2018;98:1294. 29953873 10.1016/j.neuron.2018.06.011 PMC6048952

[102] Mahan TE, et al. Selective reduction of astrocyte apoE3 and apoE4 strongly reduces Aβ accumulation and plaque-related pathology in a mouse model of amyloidosis. Mol Neurodegener. 2022;17:13. 35109920 10.1186/s13024-022-00516-0 PMC8811969

[103] Sweeney MD, Ayyadurai S, Zlokovic BV. Pericytes of the neurovascular unit: key functions and signaling pathways. Nat Neurosci. 2016;19:771–83. 27227366 10.1038/nn.4288 PMC5745011

[104] Katt ME, et al. The role of mutations associated with familial neurodegenerative disorders on blood-brain barrier function in an iPSC model. Fluids Barriers CNS. 2019;16:20. 31303172 10.1186/s12987-019-0139-4 PMC6628493

[105] Shinohara M, Tachibana M, Kanekiyo T, Bu G. Role of LRP1 in the pathogenesis of Alzheimer’s disease: evidence from clinical and preclinical studies. J Lipid Res. 2017;58:1267–81. 28381441 10.1194/jlr.R075796 PMC5496044

[106] Hampel H, et al. The Amyloid-β pathway in Alzheimer’s Disease. Mol Psychiatry. 2021;26:5481–503. 34456336 10.1038/s41380-021-01249-0 PMC8758495

[107] Montagne A, et al. APOE4 accelerates advanced-stage vascular and neurodegenerative disorder in old Alzheimer’s mice via cyclophilin A independently of amyloid-β. Nat Aging. 2021;1:506–20. 35291561 10.1038/s43587-021-00073-z PMC8920485

[108] Bell RD, et al. Apolipoprotein E controls cerebrovascular integrity via cyclophilin A. Nature. 2012;485:512–6. 22622580 10.1038/nature11087 PMC4047116

[109] Lu TM et al. Pluripotent stem cell-derived epithelium misidentified as brain microvascular endothelium requires ETS factors to acquire vascular fate. Proc Natl Acad Sci U S A. 2021;118:e2016950118. 10.1073/pnas.2016950118 PMC7923590 33542154

[110] Lippmann ES, Azarin SM, Palecek SP, Shusta EV. Commentary on human pluripotent stem cell-based blood-brain barrier models. Fluids Barriers CNS. 2020;17:64. 33076946 10.1186/s12987-020-00222-3 PMC7574179

[111] Nishihara H, et al. Advancing human induced pluripotent stem cell-derived blood-brain barrier models for studying immune cell interactions. FASEB J. 2020;34:16693–715. 33124083 10.1096/fj.202001507RR PMC7686106

[112] Praça C, et al. Derivation of brain capillary-like endothelial cells from human pluripotent stem cell-derived endothelial progenitor cells. Stem Cell Rep. 2019;13:599–611. 10.1016/j.stemcr.2019.08.002 PMC6829749 31495714

[113] Alcendor DJ. Interactions between amyloid-Β proteins and human brain pericytes: implications for the pathobiology of Alzheimer’s disease. J Clin Med. 2020;9:1490. 32429102 10.3390/jcm9051490 PMC7290583

[114] Alcendor DJ. Human vascular pericytes and cytomegalovirus pathobiology. Int J Mol Sci. 2019;20:1456. 30909422 10.3390/ijms20061456 PMC6471229

[115] Girolamo F, et al. Neural crest cell-derived pericytes act as pro-angiogenic cells in human neocortex development and gliomas. Fluids Barriers CNS. 2021;18:14. 33743764 10.1186/s12987-021-00242-7 PMC7980348

[116] Trost A, et al. Brain and retinal pericytes: origin, function and role. Front Cell Neurosci. 2016;10:20. 26869887 10.3389/fncel.2016.00020 PMC4740376

[117] Trost A, et al. Neural crest origin of retinal and choroidal pericytes. Invest Ophthalmol Vis Sci. 2013;54:7910–21. 24235018 10.1167/iovs.13-12946

[118] Fu J, Liang H, Yuan P, Wei Z, Zhong P. Brain pericyte biology: from physiopathological mechanisms to potential therapeutic applications in ischemic stroke. Front Cell Neurosci. 2023;17:1267785. 37780206 10.3389/fncel.2023.1267785 PMC10536258

[119] Faal T, et al. Induction of mesoderm and neural crest-derived pericytes from human pluripotent stem cells to study blood-brain barrier interactions. Stem Cell Rep. 2019;12:451–60. 10.1016/j.stemcr.2019.01.005 PMC6409424 30745035

[120] Shin Y, et al. Blood-brain barrier dysfunction in a 3D in Vitro Model of Alzheimer’s Disease. Adv Sci. 2019;6:1900962. 10.1002/advs.201900962 PMC6794630 31637161

[121] Niemitz E. Isogenic iPSC-derived models of disease. Nat Genet. 2013;46:7–7.

[122] Kawatani K, et al. A human isogenic iPSC-derived cell line panel identifies major regulators of aberrant astrocyte proliferation in Down syndrome. Commun Biol. 2021;4:730. 34127780 10.1038/s42003-021-02242-7 PMC8203796

[123] Ryan SD, et al. Isogenic human iPSC Parkinson’s model shows nitrosative stress-induced dysfunction in MEF2-PGC1α transcription. Cell. 2013;155:1351–64. 24290359 10.1016/j.cell.2013.11.009 PMC4028128

[124] de Leeuw SM, et al. E3, and E4 differentially modulate cellular homeostasis, cholesterol metabolism, and inflammatory response in isogenic iPSC-derived astrocytes. Stem Cell Rep 17. 2022;APOE2:110–26. 10.1016/j.stemcr.2021.11.007 PMC8758949 34919811

[125] Zhao J, et al. APOE4 exacerbates synapse loss and neurodegeneration in Alzheimer’s disease patient iPSC-derived cerebral organoids. Nat Commun. 2020;11:5540. 33139712 10.1038/s41467-020-19264-0 PMC7608683

