# Supplementary material for: iPSC-derived blood-brain barrier modeling reveals APOE isoform-dependent interactions with amyloid beta
Source: Fluids Barriers CNS. 2024 Oct 11;21:79. doi: 10.1186/s12987-024-00580-2 (PMC11468049; doi:10.1186/s12987-024-00580-2)
Supplement: Supplementary file 1 — Supplementary Material 1 [file 12987_2024_580_MOESM1_ESM.docx]

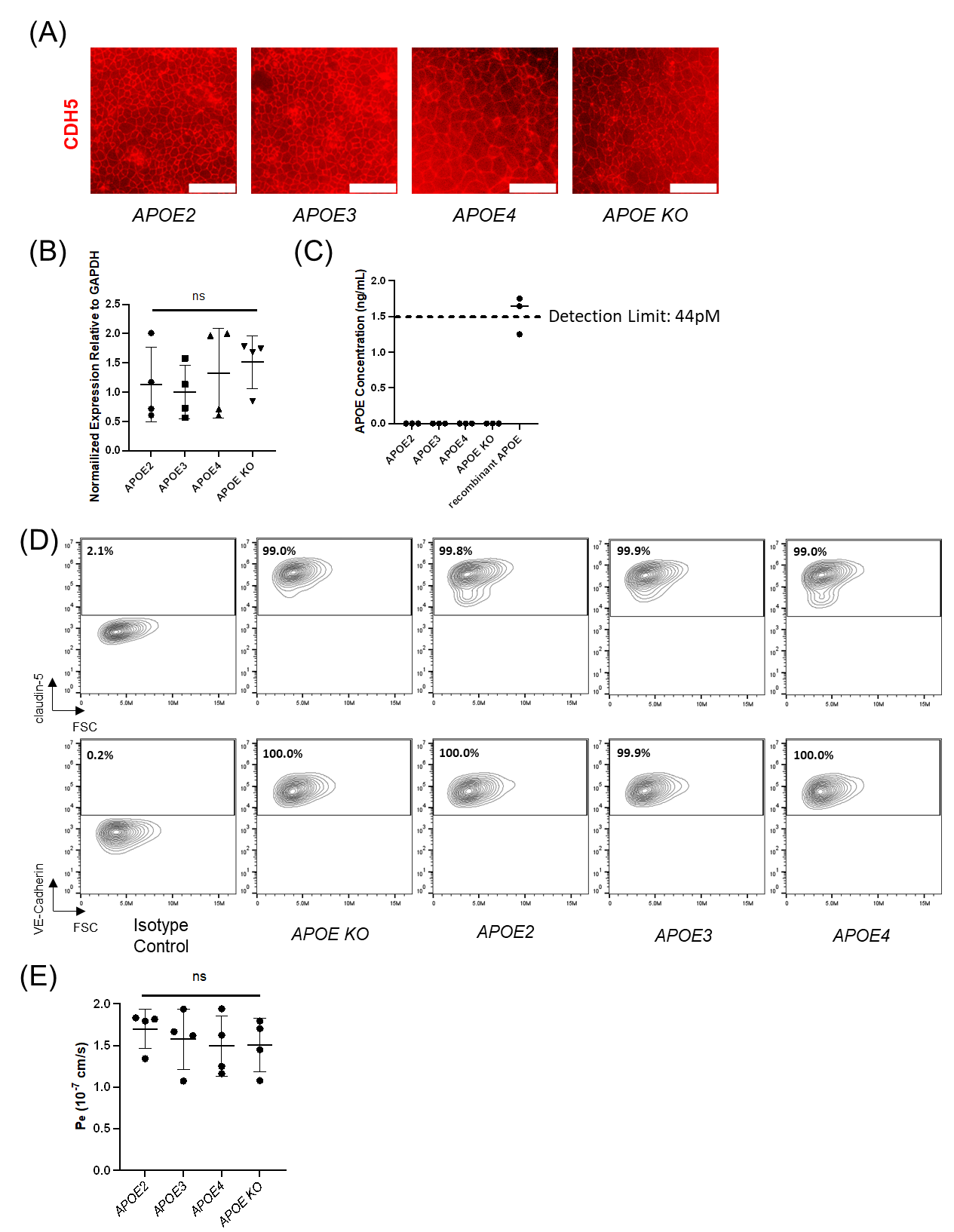


**Extended Figure 1. iPSC-derived BMEC-like cells expressing different APOE isoforms have similar protein expression and function.**

1. Immunocytochemistry analysis of VE-cadherin (CDH5) expression in Day 10 isogenic iPSC-derived BMEC-like cells Scale bars: 100 μm.
2. RT-qPCR analysis of *APOE* transcript expression in Day 10 isogenic BMEC-like cells (n=4 differentiations each). Data are reported as mean ± standard deviation. Data is normalized to the *APOE3* condition. ns: p-value>0.05 in one-way ANOVA analysis.
3. Quantification of APOE concentration by ELISA in cell culture media conditioned by Day 10 isogenic BMEC-like cells for 24 hours. APOE concentrations were not detectable in the conditioned media by ELISA, which has a detection limit of 44pM using recombinant APOE3.
4. Flow cytometry analysis of claudin-5 and VE-Cadherin expression in isogenic BMEC-like cells. FSC: forward scatter.
5. Permeability of sodium fluorescein across a monolayer of isogenic BMEC-like cells. (n=4 wells of independent differentiations for each condition). Data are reported as mean ± standard deviation. ns: p>0.05 in one-way ANOVA analysis.


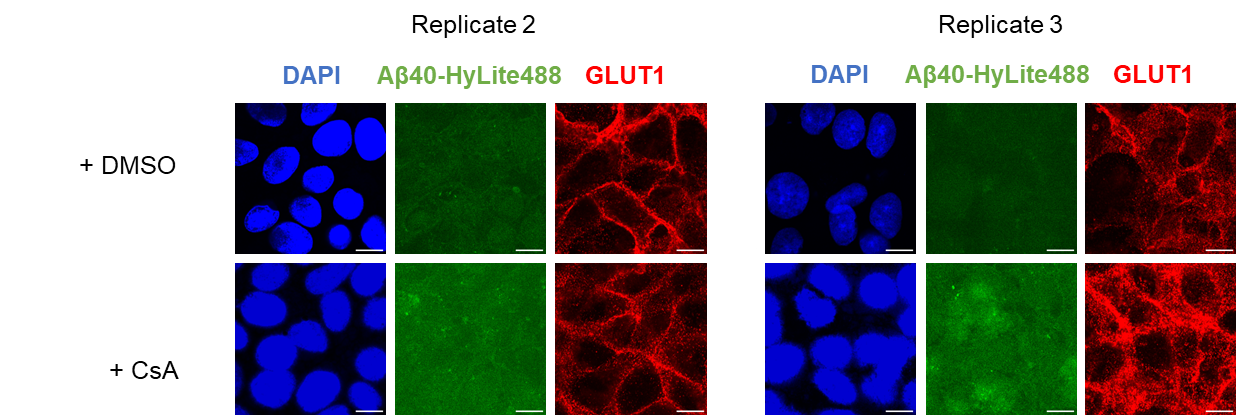


**Extended Figure 2. P-glycoprotein-mediated pathways are involved in Aβ transport by iPSC-derived BMEC-like cells.**

Confocal microscopy images to examine accumulation of intracellular Aβ40-Hylite488 with and without P-gp inhibition by CsA. Confocal microscopy images were taken at the z-slice where DAPI nuclear staining was visible. GLUT1 can be found both at the cell surface and junctions in BMEC-like cells and its immunolabeling was included to help visualization of the cell junctions at the same z-plane. Shown here are replicates 2 and 3 of Figure 2F. Scale bars: 10 μm.

**
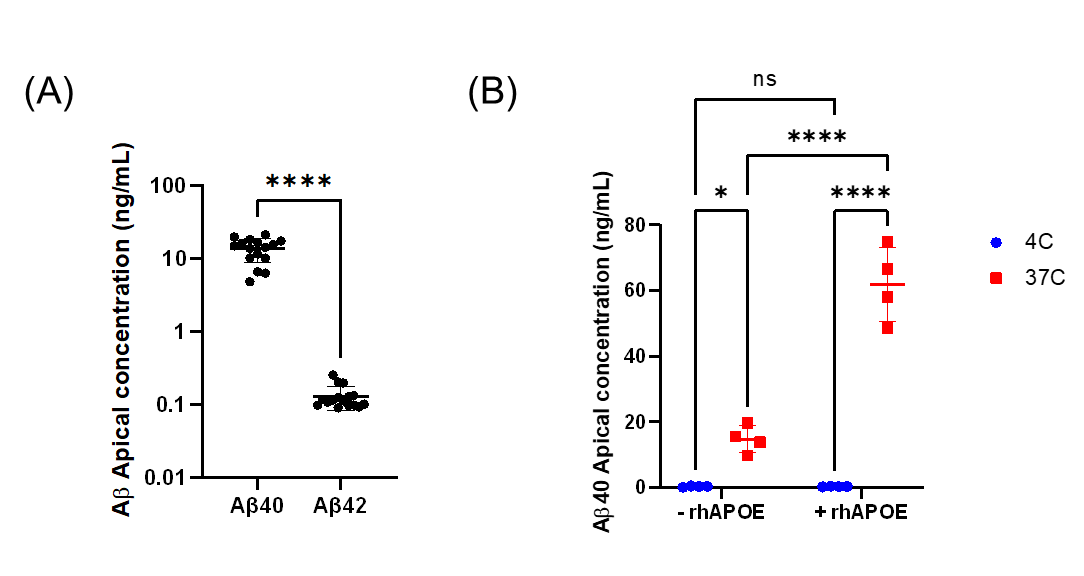
**

**Extended Figure 3. Aβ transport by iPSC-derived BMEC-like cells is transcellular.**

1. Transport of Aβ40 or Aβ42 by BMEC-like cells to the apical chamber after 3 hours of incubation quantified by ELISA. Transport data across isogenic *APOE* genotypes presented in Figure 3B and 3D were aggregated by Aβ isoform studied. ****: p<0.0001 in Student’s t-test. Data are reported as mean ± standard deviation.
2. Differential accumulation of Aβ40 by *APOE3* BMEC-like cells in the apical chamber, in the presence or absence of 500 nM rhAPOE3 in the media in the basolateral chamber, after 3 hours of incubation at 4 °C or 37 °C quantified by ELISA. (n=4 wells of independent differentiations for each condition) *: p<0.05, ****: p<0.0001 in one-way ANOVA followed by Tukey’s test. Data are reported as mean ± standard deviation.
